# Development of Dietary Knowledge and Adherence Questionnaires for Lebanese Adolescents and Their Parents

**DOI:** 10.3390/ijerph17010147

**Published:** 2019-12-24

**Authors:** Liliane Said, Jessica S. Gubbels, Stef P. J. Kremers

**Affiliations:** 1 Department of Health Promotion, NUTRIM School of Nutrition and Translational Research in Metabolism, Faculty of Health, Medicine, and Life Sciences, Maastricht University, Maastricht 6200, The Netherlands; jessica.gubbels@maastrichtuniversity.nl (J.S.G.); s.kremers@maastrichtuniversity.nl (S.P.J.K.); 2 Department of Nutrition and Food Sciences, Faculty of Arts and Sciences, Lebanese International University, Bekaa, Lebanon

**Keywords:** dietary knowledge, dietary adherence, questionnaire, feasibility, reliability, validity, adolescents, parents, nutrition, Lebanon

## Abstract

The availability of practical tools to assess dietary knowledge and adherence is essential to evaluate the effectiveness of dietary interventions. The aims of this paper were to develop reliable dietary knowledge and adherence questionnaires, suitable for Lebanese adolescents and their parents, and to estimate the feasibility of conducting studies involving such participants in the school-based setting. Eight Lebanese high schools participated in this study (involving 220 adolescents aged 15–18 years). Self-administered dietary knowledge and adherence questionnaires (the Dietary Knowledge Questionnaire (DKQ) and the Dietary Adherence Questionnaire (DAQ), respectively) were completed by the high school students and their parents. A 24 h recall was additionally administered for the adolescents by a dietitian and a trained interviewer at school, in order to validate the adolescents’ answers in the DAQ. The cognitive interview method was used to qualitatively evaluate the questionnaires. The resulting Cronbach’s alpha ranged from 0.61 to 0.78 for the adolescent questionnaires and from 0.46 to 0.89 for the parental ones. In addition, 23 items (out of 25) of the adolescent DAQ matched with the administered 24 h recall. A significant negative correlation was found between the knowledge score (DKQ) and the unhealthy items of the adolescent DAQ. There was a significant positive correlation between the DKQ of the parents and the knowledge score of their children. This is the first study of dietary questionnaires involving Lebanese high school students from different regions, while also including their parents.

## 1. Introduction

Pediatric obesity is the most common childhood nutritional disorder in the world [1]. This global epidemic is the cause of many physical complications, such as diabetes mellitus, metabolic syndrome, dyslipidemia, cardiovascular diseases, obstructive sleep apnea and hypertension, as well as psychosocial complications such as low self-esteem, higher rates of anxiety disorders, decreased health-related quality of life (QOL) and decreased educational and financial attainment [2,3]. These health consequences constitute a serious economic burden. The incremental lifetime direct costs from the perspective of a 10-year-old obese child relative to a normal weight child reaches an average of $16,000 [4]. Obesity is preventable, however [5]. According to the World Health Organization (WHO) [5], over 340 million children and adolescents aged 5–19 years were overweight or obese in 2016 worldwide. Lebanon, a Mediterranean country in the Middle East, is no exception, as 30.8% of adolescents aged 12–19 years are overweight and 10.3% are obese [6].

Since childhood obesity carries over into adulthood [5], it is important to direct all of our efforts to preventing this problem early on. However, there are no specific programs to prevent obesity in Arab countries [7]. There are some activities promoting specific healthy behaviors in some of these countries, but they have limited effects [7].

There are many behavioral factors related to pediatric overweight and obesity. It is well known that overeating and a sedentary lifestyle are the main contributors [8]. During the last five decades, most Arab countries encountered important challenges related to demographic and socioeconomic factors, and health status [7]. They are also facing a nutrition transition characterized by replacing traditional diets with high fat and processed foods [7]. In Lebanon specifically, high consumption of fast food and sugar-sweetened beverages have been found to be associated with higher odds of overweight [6]. Higher intakes of milk, dairy products, fruits and vegetables, daily breakfast consumption and decreased sedentary time were associated with significantly lower odds of overweight and obesity [6]. Identification of these determinants of overweight helps guiding the development of tools to detect these behaviors.

Both body mass index (BMI) and eating habits are strongly influenced by urbanization [9,10]. From 1985 to 2017, the proportion of the world’s population living in urban regions increased by 14%, and over the same period of time the mean BMI increased by 2.1 kg/m^2^ in women and by 2.2 kg/m^2^ in men [10]. In low to middle income countries, rural areas were previously characterized with a lower BMI compared to the cities, but the urbanization of rural life (due to the mechanization of agriculture, transport development and the availability of processed food) contributes to the increasing BMI of the rural population [10]. In Lebanon, living in the capital Beirut seems to be associated with higher odds of overweight (compared to adolescents residing in other governorates) [6], for reasons that have not been empirically studied to date. It is important to examine potential differences in eating patterns, to be able to initiate the targeted measures to avoid adverse health outcomes [11]. However, to our knowledge, no previous studies have compared the difference in dietary patterns between Lebanese adolescents living in urban versus rural regions.

Identifying the percentage of the population who follows the dietary guidelines [12] for specific food groups will guide prevention strategies to improve dietary quality and reduce the prevalence of obesity [13]. Nutritional recommendations cover a broad range of intake behaviors (e.g., water intake, fiber intake, fruit and vegetable consumption). Therefore, gaining insight on knowledge regarding specific topics allows us to elucidate the areas which require attention [14], resulting in nutrition interventions that are more specific and more efficient. Dietary knowledge is one of the few modifiable determinants influencing nutritional behavior [15]. People with higher dietary knowledge levels are almost 25 times more likely to consume sufficient amounts of fruits, vegetables and fat, compared to those with a lower level of dietary knowledge [16]. Hence, increasing knowledge will eventually improve the diet of target populations [17]. However, knowledge is not the only factor to take into consideration. It is also well known that the family exerts a major influence on adolescents’ eating behaviors [18]. Parents play a key role in the food environment of their children [19]. Parental nutritional knowledge has previously been found to be positively associated with adequate intakes of dairy products, fruits, vegetables and meat, among their children [20]. However, the association between the parents’ dietary adherence and knowledge and those of their children has not yet been studied in Lebanon. Knowing the extent of influence of Lebanese parents on their children’s eating behavior will guide future interventions to promote healthy eating and to prevent obesity.

Before planning any program aimed at improving the dietary habits of adolescents, it is crucial to identify the necessary tools to monitor and assess intervention effects. Information related to dietary patterns can be obtained from tools measuring nutrient intake (e.g., food records, 24 h recall, etc.), but they are mostly complicated, time consuming and expensive [21]. In addition, such tools often present a high subject burden and methodological concerns when used for school-aged children [22], whereas self-administered questionnaires present no interviewer-related bias, are time-saving, and permit more careful responding (since respondents are not hurried) [23]. Questionnaires can be a feasible, reliable and valid method to evaluate nutrition knowledge and the correlation between dietary awareness and eating behavior [24].

There is a paucity of validated dietary knowledge and adherence questionnaires for Lebanese adolescents. Previous dietary knowledge questionnaires were often designed specifically for a particular study, and lacked psychometric validation [16]. In addition, the few available studies involving Lebanese youth did not describe the details of the process for developing such questionnaires. In addition, despite the difference between urban and rural regions as described above, previous studies often did not include participants living in rural regions [25]. This highlights the need to develop reliable and valid, user-friendly and inexpensive tools to assess levels of dietary knowledge and adherence among Lebanese adolescents living in urban and rural areas.

The current study assessed the psychometric properties of new instruments to measure the dietary knowledge and adherence to the nutritional guidelines of Lebanese adolescents. The aims of this paper are to describe the step-by-step process of developing these questionnaires for Lebanese adolescents living in urban and rural areas and their parents, to analyze their feasibility and internal reliability in the target population, and to examine the correlation between parental and adolescent scores.

## 2. Materials and Methods

**Figure 1:**
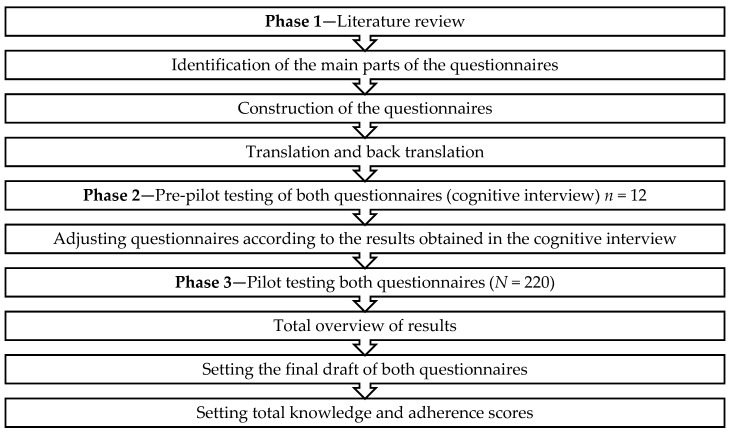
Development phases of dietary knowledge and adherence questionnaires.

We developed two questionnaires: The Dietary Knowledge Questionnaire (DKQ), to assess the level of dietary knowledge of Lebanese adolescents and their parents, and the Dietary Adherence Questionnaire (DAQ), to assess the level of adherence to nutritional guidelines [12]. Both questionnaires were developed following the same steps (see Figure 1).

### 2.1. Phase 1—Literature Review

First, a thorough literature review of dietary knowledge, general dietary recommendations for adolescents and dietary behaviors linked to overweight and obesity, was done to identify the main parts of the questionnaires. Second, the format of both questionnaires was determined (e.g., type of questions, method of administration, duration, language, etc.). Both questionnaires are self-administered structured ones with closed questions. All questions were designed to suit both target groups (adolescents and adults) and participants with different socioeconomic backgrounds.

Particular attention was paid to the first few questions, as they influence the respondent’s attitude and cooperation [26]. The first page of both questionnaires included statements summarizing the purpose of the study and confirming the consent to participate once the questionnaire is completed and submitted.

Both questionnaires were administered during classes and in Arabic. They were designed to be convenient for Lebanese adolescents and their parents. It should be possible for 20 to 35 students to fill out the questionnaires during a class session lasting 40–50 min. This explains the reason behind choosing a self-administered format, closed questions and a relatively small number of answer options. To make sure that the selected items suited the Lebanese population and culture, some were taken from existing questionnaires (see below), while others were added or adapted to the Lebanese eating culture.

The third step involves translation and back translation. Both questionnaires were originally developed in English by a dietitian with practical experience in nutrition in Lebanon, then translated into Arabic, and back translated to English. The initial translation and back-translation were done by two different individuals. To avoid bias, the back-translator was not aware of the concepts the questionnaires intend to measure [27].

#### 2.1.1. Dietary Knowledge Questionnaire

The Dietary Knowledge Questionnaire (DKQ) aims to assess the level of dietary knowledge of Lebanese adolescents (see Supplementary Materials S1 and S2), and their parents (see Supplementary Materials S3 and S4). It was based on the Nutrition Knowledge Questionnaire (NKQ) developed by Parmenter and Wardle [17], which meets the criterion for construct validity, and has a total Cronbach’s alpha of 0.97, reflecting a high internal reliability. It was adapted to suit the Lebanese culture: for instance, Lebanese food items, such as Akawi cheese and labneh, replaced foods less common in Lebanon, like edam cheese. The selected items were meant to be representative of a broad range of dietary knowledge [17]. The questions ask about very basic and general nutrition information to assess whether the participants have a minimum level of knowledge, allowing them to make decisions regarding their food intake.

The DKQ consists of five parts and a total of 56 items (including questions and sub-questions) and takes 15–20 min to fill out. Some questions are divided into additional sub-questions, making 40 sub-questions in total. Each question has three to five answer options, and only one correct answer per question/sub-question. A “do not know” option was added to all questions.

The five parts are: (1) Knowledge of dietary recommendations, to determine if participants are familiar with the current ones; (2) knowledge of nutrient sources, to determine whether participants know if the indicated food items are good or bad sources of the nutrient in question; (3) knowledge of common misconceptions in nutrition, to determine whether participants are aware of some of those related to nutrition; (4) using knowledge of nutrition to make dietary choices, to determine whether participants are able to choose the healthiest option when given different food choices; (5) knowledge of associations between nutrition and diseases, to determine if the participants are aware of the correlation between eating habits and health. The original NKQ questionnaire included the same parts [17], but the “understanding of terms” part was renamed “knowledge of common misconceptions in nutrition”, although the questions remained the same. As more than 92.7% of young adults relies on online resources to obtain nutrition information and only 4.7% refers to healthcare professionals, it is important to verify if the received information is correct [28].

Answers were coded as follows: zero for the wrong answer or if the participant did not know the answer, and one point for the correct answer.

The weighting of the various items is indicated in Appendix A. The total knowledge score was calculated by summing up the points on all items. Thus, the maximum possible score is 56 and the minimum is zero. The same DKQ was administered to both the parents and their children.

#### 2.1.2. Dietary Adherence Questionnaire

The Dietary Adherence Questionnaire (DAQ) (see Supplementary Materials S5 and S6) was based on the Schools Physical Activity and Nutrition Survey (SPANS) years 8 and 10 student questionnaire [28]. The questions and answer options were modified to lower the burden for participants by making them shorter, to avoid response fatigue and inaccurate results [29]. It is a self-administered questionnaire with close-ended questions, and takes about 20 min to complete. It consists of 30 questions and is divided into four parts: (1) food choices, related to the consumption of specific food groups like lean meats, sweets, etc.; (2) eating habits, related to meal pattern consumption such as the number of meals, snacks and breakfast consumption; (3) physical activity (PA) and screen-viewing time; (4) smoking. The number of options per question ranges from two to six.

Even though the DAQ included one question regarding smoking and four related to engaging in PA and screen time, it was still called the Dietary Adherence Questionnaire because those questions were added to examine the correlation of diet with smoking and PA to identify potentially clustered behaviors. Based on a cross-sectional study of Brazilian adolescents, a significant positive correlation was found between smoking and a high intake of sweets, low fruit intake and high intake of soft drinks [30]. The questions regarding PA and screen-viewing time were added because studies have shown that active individuals tend to have healthier diets [31]. The questions and scoring system were identical for both the parents and their children, except for the question about PA in school, which was omitted as irrelevant from the parents’ DAQ (see Supplementary Materials S7 and S8).

This questionnaire was designed to assess each question separately by checking, for instance, whether the participant eats high fat meat or lean meat, or eats breakfast or not. However, to facilitate the evaluation of the overall dietary behavior, instead of looking at each question separately, the total adherence index was created. Therefore, it presents a useful, user-friendly and rapid tool for health promotors to detect unhealthy eating behavior, potentially leading to overweight or obesity and correcting it. It also allows us to compare the answers before and after an intervention.

Scoring was zero for “No, I did not eat any of the foods listed above yesterday”, one point for “Yes, I ate it once”, two points for “Yes, I ate it twice”, and three points for “Yes, I ate it three times or more”. In the question related to breakfast consumption, the option “yes” was equal to two points, while “no” was equal to zero, as breakfast cannot be consumed more than once a day, as well as to emphasize the importance of this meal. In questions 26 and 27, related to PA practice, “yes” was equal to one point and “no” to zero. The weekly duration of PA practiced was coded as follows: zero for “0 h”, one point for “1 h”, two points for “2–4 h” and three points for “more than 5 h”. As for smoking, “yes” was equal to two points, and “no/stopped smoking” was equal to one point. The question related to screen-viewing time (duration spent watching television, using phones or tablets, or playing video games) was removed to improve reliability (increased Cronbach’s alpha).

Next, to simplify the use of this questionnaire in interventional studies, and to be able to assess the overall adherence to dietary guidelines instead of looking at each question separately, a total adherence index was developed by dividing the score of healthy items by the score of unhealthy items:

Total adherence index = Healthy items score/Unhealthy items score

If the score is above 1, then the healthy food choices and habits outweigh the unhealthy ones, and if the score is below 1, there are more unhealthy choices compared to healthy choices (see Table 1 and Appendix B). All individuals started with one point on the unhealthy score to avoid zero as a denominator. The maximum possible index score is 37 (reflecting a total adherence to dietary guidelines) and the minimum score is zero. The items related to the number of snacks and juices consumed were removed from the calculation of the index score, as it is hard to distinguish whether the snack itself is healthy or unhealthy. For instance, an apple is not the same as soft drinks. The same goes for natural juices: to avoid controversy about whether to consider the item healthy or unhealthy, it was not included in the index. Thus, a maximum of 37 points for the healthy items and a maximum of 38 points for unhealthy items is obtained.

It is important to note that the DAQ does not investigate the reasons behind the participants’ actions and attitudes.

### 2.2. Phase 2—Cognitive Interview

The cognitive interview method [32] was selected to test the clarity and interpretation of the Arabic version of the questionnaires. According to Miller [33], large sample sizes are not necessary at this stage because problems in questionnaires are supposed to be revealed immediately. Therefore, 12–25 cases are sufficient to pre-test questionnaires, although sample sizes are often influenced by the survey schedule, leading to a typical sample of 5–15 participants [33].

A group of 12 students was asked to fill out both questionnaires (DKQ and DAQ) and to add a (+) or a (−) next to the questions they liked or did not like for different reasons. For instance, they could add a (−) next to the questions they did not understand or if something was not clear, and a (+) sign next to the comprehensible questions. After filling out the questionnaires, each student handed them back and discussed their comments with the interviewer (see Table 2). Adjustments were made to the final draft of the questionnaires according to the results obtained during the cognitive interviews, before testing both questionnaires on a larger sample size.

### 2.3. Phase 3—Pilot Testing the Questionnaires

#### 2.3.1. Inclusion Criteria and Sampling Method

**Figure 2:**
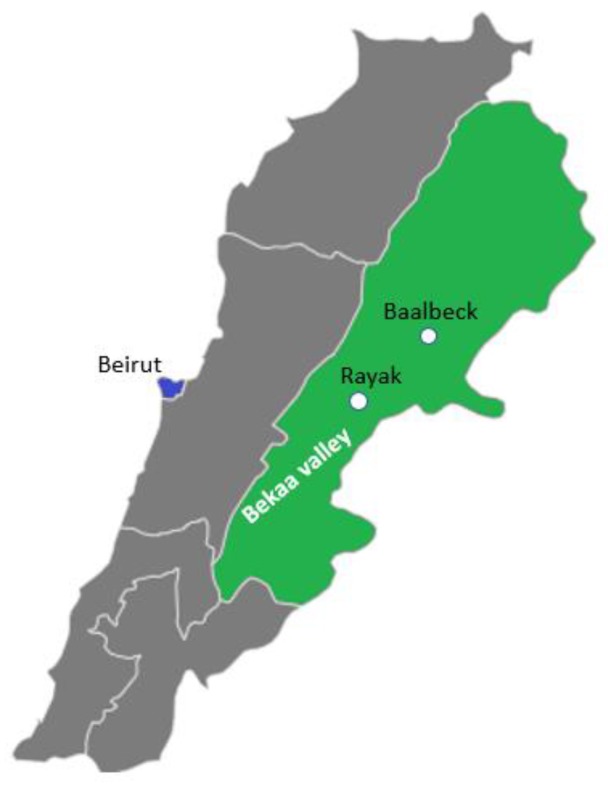
Map of Lebanon.

Lebanese public and private high schools located in Beirut, Baalbeck and Rayak were included, thus covering both urban and rural regions. The urban region refers to Beirut, the capital and largest city of Lebanon, and the rural region refers to Baalbeck and Rayak, located in the Bekaa region, which is considered Lebanon’s most important farming region (see Figure 2).

Even though Baalbeck is a small city, it is considered part of the rural area along with Rayak, as schools in Baalbeck include many children coming from the villages nearby. In addition, no international fast-food chains, shopping malls or buses (connecting different locations in the same town) are located in Baalbeck. There are only some local restaurants and coffee shops.

As few studies involved Lebanese adolescents, assessing the feasibility of conducting research in Lebanese school-settings is one of the first key learnings of the current study. This includes experiences with school and participant recruitment, questionnaire administration in classes, data collection scheduling during class time, and facing challenges and opportunities in the implementation of the study protocol.

Several inclusion criteria for the participating adolescents were used: (1) being Lebanese and enrolled in Lebanese public or private high schools located in Beirut, Baalbeck and Rayak; (2) aged 15–18 years; (3) being fully capable (cognitive, psychiatric and physical ability) of communicating (as reported by parents or by school administration); (4) not having any chronic or genetic disease (such as chronic kidney diseases or other diseases, as reported by parents or by school administration). Lebanese high schools are either public or private. Public high schools are managed by the Lebanese Ministry of Education and Higher Education, while private schools are managed by individuals or organizations. Due to the higher tuition fees in private schools, Lebanese families with higher incomes tend to enroll their children in private schools, while children from lower socioeconomic groups more often attend public schools. In this study, both types of schools were taken in consideration to include adolescents from different socioeconomic backgrounds.

The sampling method was nonselective, meaning that all participants meeting the inclusion criteria and present at the moment of data collection were selected. All subjects gave their informed consent for inclusion before they participated in the study. The questionnaire stated clearly that once it was filled in and handed in, this meant that the adolescent accepted and assented to participate in the study. Consent was also orally confirmed by the PI before administering the questionnaire. A similar assent statement was also included in the parents’ version of the questionnaires. The study was conducted in accordance with the Declaration of Helsinki, and the protocol was approved by the Lebanese Ministry of Education and Higher Education (3/15465; date: 24 October 2016).

The total number of adolescents enrolled in high schools located in Beirut (urban area) is 12,983 [34]. The number of public schools located in Beirut is 18 (obtained from the Lebanese Ministry of Education and Higher Education). However, the total number of private high schools in this region is not reported. The number of adolescents enrolled in high schools located in Bekaa (Baalbeck, Rayak and other towns and villages) is 15,843 [34]. The number of public high schools located in Baalbeck and Rayak is four, the number of private schools is not reported. For the current study, a total of seven schools were recruited: four public (one from Beirut, two from Baalbeck and one from Rayak) and three private (one from each city/town) schools.

According to Hulley et al. [35] and taking into consideration that this observational study involves correlations as the principal form of analysis, the total sample size is calculated as follows:

Total sample size = N= [(Zα + Zβ)/C]2 + 3; taking into consideration:

- α = 0.05; Zα = 1.96; β = 0.2; Zβ = 0.842
- r = 0.3 (according to Asaad et al. [36], the obtained correlation between fruits and vegetable servings, high sugar foods and high fiber foods obtained from the perceived dietary adherence questionnaire and the 24 h recalls were 0.30, 0.40 and 0.46, respectively. Other correlations were not considered, as they were irrelevant in our case, e.g., low glycemic index foods)
- C = 0.5 × ln [(1 + r)/(1 − r)]

This results in a minimally required sample of N = 85 participants. The actual obtained sample size in the current study was 220.

#### 2.3.2. Questionnaire Administration and Data Collection

Data collection took place between January and May 2017. A total of seven high schools were included. School administrations assigned one session per class during the normal schedule (40–50 min). The interviewers visited each class, explained the purpose of the study in detail, gave the necessary instructions to fill out the DKQ and DAQ questionnaires, and distributed and collected them. In addition, a 24 h dietary recall was administered to each student by the interviewers during the same session. The 24 h recalls were unstructured, although a stepwise protocol was used. A blank sheet was filled by the interviewer based on what the participants answered to the question concerning what they ate in the previous 24 h. In the next step, the interviewers asked about the missing information, such as the portion sizes, brand names of food items and cooking methods (grilled, boiled, fried, etc.). At the end, the interviewers went over the answers one more time, to make sure that the adolescents did not forget anything. The recall was used to compare the DAQ filled in by the students with the DAQ filled in by an expert based on the 24 h recall, so as to check the validity of the DAQ answers related to food and meal consumption. In addition, the parents’ DKQ and DAQ were handed to the children, for their parents to complete at home. After one to two weeks, the completed parental questionnaires were collected at the schools.

### 2.4. Statistical Analyses

Data were entered and analyzed using the Statistical Package for Social Sciences version 25.0 (SPSS Inc., Chicago, IL, USA). *p*-values of <0.05 were considered statistically significant. Descriptive statistics were used to analyze the participant characteristics. Cronbach’s alpha test was used to assess the internal consistency and reliability of the questionnaires. Coefficients from 0.5 to 0.75 suggest moderate reliability, and values >0.75 reflect good reliability [37].

Independent t-tests were employed to determine significant differences between subgroup means (boys vs. girls, urban vs. rural and public vs. private). Pearson’s correlations were used to explore potential relations between knowledge and the adherence scores of adolescents and parents. One-Way analysis of variance (ANOVA) Post Hoc was used to determine any differences between the three study locations.

## 3. Results

### 3.1. Cognitive Interview

A total of 12 adolescents was asked to fill out both questionnaires (DKQ and DAQ) and comment on them in a cognitive interview. These opinions were divided into three categories: (1) language problems (e.g., not knowing the meaning of a word); (2) logical problems (interpreting questions the wrong way); and (3) general feedback (e.g., positive feedback; not shown). The comments and subsequent adjustments to the questionnaires are listed in Table 2.

### 3.2. Demographic Characteristics

A total of 220 adolescents, recruited from seven high schools, submitted both questionnaires. As for the parents, 108 (49%) returned the DKQ and 89 (40.5%) filled out the DAQ. Their demographic characteristics are shown in Table 3.

### 3.3. Feasibility

All adolescents agreed to participate, and they were positively engaged. They were able to fill out both questionnaires during the assigned time. The presence of a teacher in class was experienced as favorable, as the adolescents were more disciplined and worked faster.

### 3.4. Internal Reliability

The internal reliability reflects how well the individual items of the scores fit together and assess the same construct [22]. Values for Cronbach’s alpha ranged from 0.46 to 0.89 (see Table 4).

### 3.5. Dietary Knowledge and Adherence Scores

Adolescents scored an average of 30.7 (SD ± 6.9) on the DKQ, ranging from 7 to 50, and 34.54% answered more than half of the questions incorrectly. The mean total adherence index score was 2.1 (SD ± 1.5), ranging from 0.2 to 12, and 10% of adolescents obtained a score below 1. In terms of demographic characteristics such as age, gender and type of school, there was no significant difference, either for the knowledge score or for the total adherence score between groups (see Table 5). There was a significant difference for the knowledge score between urban and rural regions (*p* < 0.01). When comparing the three locations separately, there was a significant difference for the total knowledge and adherence scores between them (see Appendix D). Post-hoc analyses revealed that the difference was significant between Beirut and Baalbeck (*p* < 0.05). Parents had a mean of 32.4 (SD ± 10.0) on the DKQ, ranging from 2 to 49, and there were no significant differences between the groups based on demographics. Detailed responses of both questionnaires are shown in Appendix C and Appendix E.

### 3.6. Correlations between Scores

The DAQ of the parents was not included in the correlation analyses, as its Cronbach’s alpha was low (<0.50). There was no statistically significant correlation between the knowledge score of adolescents and the corresponding total adherence index (Table 6). However, a significant negative correlation (r = 0.158, *p* = 0.02) was found between the DKQ and the unhealthy items score of the DAQ.

There was a significant positive correlation (r = 0.359, *p* < 0.001) between the knowledge score of the parents and the knowledge score of their children. The parental knowledge score further correlated positively with the total adherence score of the children (r = 0.242, *p* = 0.02). A significant positive correlation was found between the unhealthy items and the healthy items of DAQ (r = 0.33, *p* < 0.001).

When comparing the DAQ items completed by the adolescents and those by the dietitian based on the 24 h recall, we found a significant, positive correlation between all the items related to food intake and meal consumption (questions 1–25) in the DAQ and their correspondents based on the 24 h recall, except for two items (question 8—skimmed milk and question 18—diet soft drinks) (see Appendix F).

## 4. Discussion

Overweight and obesity are multifactorial and largely preventable conditions [38], affecting more than 40% of Lebanese adolescents [6]. However, prior to planning any health promotion program targeting this particular age category, it is necessary to have reliable tools to assess the current levels of dietary knowledge and adherence to determine the effectiveness of such interventions later on. To our knowledge, studies assessing the relationship between dietary knowledge and adherence are scarce [25], particularly in the Middle East. Correspondingly, there is a paucity of reliable Arabic questionnaires measuring dietary knowledge and adherence.

Few studies have described the detailed process of developing such questionnaires, and none of them investigated the potential correlation between the dietary knowledge and adherence of the parents and of their children.

Many dietary questionnaires do not include physical activity questions, even though it is an essential part of energy expenditure [39]. Nor are all methods of dietary assessment (e.g., 24 h recall, diet records, etc.) feasible for administration in school settings due to their high cost and respondent burden [39]. The aim of the present paper was to describe the step-by-step process of developing dietary knowledge and adherence questionnaires suitable for Lebanese adolescents and their parents living in urban and rural areas, to examine their feasibility and internal validity in the target population, and to analyze the correlation between parental and adolescent scores.

### 4.1. Feasibility

As few previous studies involved Lebanese adolescents, it was important to assess the feasibility of conducting research in Lebanese school-settings (i.e., recruiting schools and participants, administering questionnaires to large groups of individuals at once, investigating potential challenges and opportunities). This step is essential, not only to guide future assessment studies, but also to plan future nutrition intervention in Lebanese schools. All adolescents agreed to participate in the study. By contrast, their parents showed low participation rates (49.0% for DKQ and 40.5% for DAQ). This suggests that involving the parents in future nutrition studies might be quite challenging, in line with previous findings [40]. There might be several reasons behind the low participation rate of the parents, including a lack of motivation to participate in research studies or events organized in schools. This was confirmed by school principals of the schools participating in the current study, reporting that many parents do not attend parents’ meetings or lectures organized by the schools. This was in line with another study conducted in Lebanese schools [40]. Some parents might not have received the questionnaires, as some adolescents reported forgetting to hand the questionnaires to their parents, even though many teachers reminded them. In addition, parents might have a lack of interest in activities that do not affect the educational attainments of the children. Besides recruitment of parents, another challenge was recruiting private schools to participate in the study. There was no difficulty in convincing public schools, since we obtained the approval of the Lebanese Ministry of Education and Higher Education, allowing direct access to public schools. The private schools had to be approached individually for participation. Similar challenges were also reported in another study involving Lebanese children aged 9–11 years [40].

As for the feasibility of administering the questionnaires, all students were able to fill in both DKQ and DAQ during the assigned time, and the interviewer was also able to collect a 24 h recall in the same session. For classes with more than 20 students, two interviewers are required to cover all participants. Also, in some cases, it was beneficial to work with the students in the presence of a teacher or teacher assistant, as they were more disciplined and worked faster then.

### 4.2. Internal Reliability

The internal reliability of the dietary knowledge questionnaires we developed for both adolescents and their parents was more than acceptable [37]. The parental version had a somewhat higher Cronbach’s alpha value, which can be explained by the fact that the current DKQ was based on a knowledge questionnaire that was originally developed for adults [17]. The internal validity of the current questionnaire is higher compared to previously developed knowledge questionnaires for younger Lebanese children (aged 9–11 years; alpha: 0.66) [25], and for older adolescents (aged 17–18 years; alpha ranged from 0.5 to 0.75) [41].

As for the dietary adherence questionnaire, the Cronbach’s alpha was moderate for both components of the total adherence score for adolescents (healthy and unhealthy items), as well as for the unhealthy items for parents. A lower Cronbach’s alpha was found for the healthy items of the adherence questionnaire of the parents, which can potentially be attributed to the small number of participants who filled in the DAQ (N = 89). Therefore, we suggest retesting it on a larger sample size in future studies. It is difficult to compare the current DAQ to similar questionnaires in other studies, because the scoring system and the target sample are often different, and Cronbach’s alpha values were not always calculated [22].

Nonetheless, a nutrition questionnaire developed for Australian children aged 10–12 years and the New Zealand Diet Quality Index for Adolescents aged 14–18 years had both a lower Cronbach’s alpha (0.5) for healthy behavior score and for measuring diet quality, respectively [22,42].

### 4.3. Dietary Knowledge and Adherence Scores

Adolescents scored a mean of 30.7 ± 6.95 for their dietary knowledge score, while their parents scored 32.4 ± 10.03, both groups scoring positive on more than half of the items on average. Even though only around 30% of adolescents answered incorrectly on more than half of the DKQ, this does not exclude the fact that some adolescents might have scored correctly by chance. The findings underline the necessity of studying the dietary adherence of adolescents within the contextual influence of dietary knowledge and of developing interventions to improve their dietary knowledge level. Another study involving adolescents aged 17–19 years showed that 27.4% had inadequate nutrition knowledge [14], which is similar to the current findings. A higher weighting was used for the nutrient sources, compared to the other parts, partly because we believe that it will be easier to understand the difference between healthy and unhealthy foods after explaining the nutrients present in different foods; the same weighting was used in the original knowledge questionnaire [17].

It is important to note that the absence of a significant difference between the dietary knowledge level of Lebanese adolescents enrolled in public vs. private schools suggests that using the same educational material and strategy when planning nutrition education is appropriate.

As for the total adherence index score, adolescents scored a mean of 2.1 ± 1.48, indicating that the healthy items outweighed the unhealthy ones. However, although only 10% had an adherence score of less than 1, this does not mean that the remaining 90% had adopted healthy lifestyles. The DAQ focuses on the frequency of consumption of a certain item and not on the portion size, as it uses a non-quantified food frequency approach [39]. Its main purpose was to compare the effectiveness of dietary interventions and to track eating patterns over time. It included short questions about the previous day, and the number of questions was limited to address the specific behaviors related to weight gain and to ease the respondent burden [39].

### 4.4. Correlations Between Scores

In addition to the reliability analyses, a validity testing of the adolescent DAQ was done by comparing results from the food choice and eating habits sections and 24 h recall. This showed significant correlations for the vast majority of the items (23 out of 25), indicating that the DAQ is a valid tool in reflecting the food choices and eating habits of Lebanese adolescents [21]. These findings differ from those in other studies. Hoelscher, Day, Kelder and Ward [39] reported that the percentage agreement between the School-Based Nutrition Monitoring questionnaire and the 24 h recall ranged from 38% to 89%, and Asaad, Sadegian, Lau, Xu, Soria-Contreras, Bell and Chan [36] reported that six out of nine items (67%) of the Perceived Dietary Adherence Questionnaire designed for diabetic patients were significantly correlated with the 24 h recall. However, the current study showed no significant correlation between the DAQ and the dietary recall for the items regarding skimmed milk and soft drinks. Perhaps the adolescents missed the word “skimmed” (for milk) and “diet” (for soft drinks), since both questions related to similar food items in the previous question (i.e., regular milk; regular soft drinks) with only a one word difference, unlike all the other questions.

The correlation between the total knowledge and total adherence index scores of adolescents was not significant. This was similar to the finding in a previous study of adolescent athletes, which also showed no correlation between nutritional knowledge and dietary intake [15]. However, the knowledge score of adolescents was strongly correlated with the unhealthy items of the total adherence score rather than with the healthy items. This suggests that when adolescents know more about nutrition, they seem to eat less unhealthily. In other words, dietary knowledge may act as an inhibitor of unhealthy behavior, but to a lesser extent as a stimulant for healthy food choices. Future studies should consider interpreting the total adherence index versus examining the healthy and unhealthy components of the DAQ separately.

Very few studies have previously investigated the correlation between dietary adherence and dietary knowledge, making it difficult to compare these results. Our findings are somewhat similar to another study reporting that a higher nutrition knowledge score was associated with a significantly higher consumption of some healthy food items and a significantly lower consumption of some unhealthy food items [43]. In addition, Kresic et al. [44] reported an association between the nutrition knowledge of university students and their dietary intake, but this relationship was significantly influenced by other factors, such as gender and university status.

The positive correlation between both adherence score components (healthy and unhealthy items) can be regarded as surprising. It might be explained by the fact that when an individual is eating excessive amounts of foods, he/she is eating unhealthy and healthy food items at the same time, especially as the traditional Lebanese diet includes both healthy (e.g., labneh, salads, etc.) and unhealthy food (e.g., Arabic sweets, high fat cheese, etc.). More studies are needed to examine these findings further.

From the present study, it seems that their parents’ knowledge affects adolescents’ food choices and practices more than their own knowledge does. Similarly, parental knowledge regarding the daily recommended servings of many food groups, has previously been found to be positively associated with adequate average child intakes of dairy products, fruit, vegetables, grains and cereals and meat [20]. This might not be surprising, because parents play a pivotal role in the development of their child’s energy intake habits (food preference, availability of energy-dense food, etc.) [45]. There was also a positive correlation between the knowledge score of the parents and that of their children.

### 4.5. Strengths and Limitations

The current study has various strengths. A systematic mixed methods approach, combining both qualitative and quantitative analyses, was used to develop and test both questionnaires. They were designed by a dietitian with practical experience in nutrition in Lebanon. Adolescents from three different areas in Lebanon with differing socioeconomic status were included in the sample (both urban and rural areas). The study also had an appropriate sample size (N = 220). According to Oldewage-Theron et al. [29], a minimum sample size of 94 adolescents is required to study nutrition knowledge and dietary intake patterns.

As for the limitations, while most of the DAQ questions were compared against the 24 h recall, this was not the case for the DKQ and the rest of the DAQ questions related to physical activity, screen-viewing time and smoking. Further validation of the DKQ and the rest of the DAQ is desirable. In addition, more girls than boys participated. This can be attributed to the higher number of girls enrolled in Lebanese schools [30]. Some Lebanese public high schools are for girls only, but there are no schools for boys only. Another limitation is the unequal number of adolescents in the urban and rural regions. Rural regions have fewer schools, but the number of students per class is higher compared to urban high schools. The unequal number between public and private school students is due to the higher number of students per school in the recruited public schools compared to private high schools.

### 4.6. Implications for Research

The current pilot study is the first one including Lebanese adolescents from both rural and urban areas, and their parents. The development of a measure of dietary knowledge and adherence designed specifically for use among Lebanese adolescents is likely to be a useful addition to existing methods of dietary assessment. Both questionnaires are inexpensive, time-saving, and present a low burden. The questionnaires can be used in epidemiological studies to assess the general knowledge level, gaps in certain areas in nutrition, food choices and eating patterns. As nutrition knowledge is a broad topic, specifying the areas to focus on can guide future nutrition interventions and education strategies in nutrition, and elucidate the hidden problems.

### 4.7. Implications for Practice

The DKQ and DAQ can be used by public health promoters and researchers when planning and assessing nutrition interventions for adolescents, and by policy makers to develop effective strategies to promote healthy eating and prevent obesity. The DAQ is helpful to track progress by looking at the total adherence index reflecting the overall dietary adherence, and then looking closer at separate items separately to identify specific targets (e.g., low fruit and vegetable intake, frequent soft drink consumption, meal skipping). In addition, both questionnaires can be used by nutrition professionals and dietitians in face-to-face counseling to evaluate levels of dietary knowledge and adherence of adolescents, and therefore specify the areas and topics to focus on when educating individuals and planning diets.

## 5. Conclusions

The Dietary Knowledge (DKQ) and Dietary adherence questionnaires (DAQ) are brief and relatively inexpensive assessment questionnaires of dietary knowledge and adherence to nutritional recommendations that can be used in Lebanon. Future testing should investigate whether these questionnaires can be used in other Arabic countries. This study paved the way for subsequent nutrition education campaigns by assessing the feasibility of conducting such interventions and providing them with useful tools to evaluate their success.

## Figures and Tables

**Table 1:** Total adherence score components for adolescents.

Items	Questions	Maximum Points
Healthy Items	1—Yesterday, did you eat meat (like chopped meat in stews), chicken breast (grilled/boiled/not fried), or fish (grilled/boiled/not fried)? 4—Yesterday, did you eat any of these foods? Labneh, shanklish, kareesha 5—Yesterday, did you eat any of these cheeses: Mozzarella, feta, akawi, baladiye, khadra? 7—Yesterday, did you drink milk or laban? 8—Yesterday, did you drink skimmed milk (reduced or 0% fat) or skimmed laban? 10—Yesterday, did you eat whole bread, oat bread, brown bread, tortillas? 12—Yesterday, did you eat beans like lentils, white beans, fava beans (do not count green beans)? 13—Yesterday, did you eat any vegetables (do not count potatoes)? 14—Yesterday, did you eat fresh fruits? Do not count fruit juice and dried fruits. 22—Yesterday, did you have breakfast? 23—Yesterday, how many meals did you eat (meals include breakfast, lunch, and dinner)? 26—Do you participate in physical education sessions at school? 27—Do you participate in any sports activity (other than physical education session at schools)? 28—How many hours per week do you exercise?	37
Unhealthy Items	2—Yesterday, did you eat fried chicken, chicken nuggets, fried fish, fried meat, hot dogs, sausage, mortadella, or ham? 3—Yesterday, did you eat chocolate cream or potato chips? 6—Yesterday, did you eat any of these foods: Cheddar cheese, gruyere, edam, goat cheese, gouda, parmesan, Roquefort, kashkawan, cream cheese, kishk? 9—Yesterday, did you eat white Arabic bread, kaak, franjeh bread? 11—Yesterday, did you eat French fries (fried potatoes)? 16—Yesterday, did you drink commercial fruit juice? 17—Yesterday, did you drink soft drinks (Pepsi, 7up, Miranda, Sprite, Coca Cola, Fanta …)? 18—Yesterday, did you drink diet soft drinks (Pepsi diet, 7up diet, Coca Cola diet, …)? 19—Yesterday, did you drink energy drinks (Red Bull, AMP, …)? 20—Yesterday, did you eat any sweets such as sweet rolls, cookies, cakes, pies, brownies, cheesecake, doughnuts? 21—Yesterday, did you eat any Arabic sweets (namoura, knefeh, halewet el jibn, znood el sitt …)? 25—Yesterday, how many times did you eat food from outside of your house? (restaurants, fast food restaurants, pizza places and cafeterias) 30—Do you smoke? (cigarettes and narjileh are included)	38

**Table 2:** List of comments reported by the adolescents and the corresponding adjustments to the questionnaires, based on the cognitive interview results.

Feedback Categories	Comments	Adjustments
1. Language Problems	DAQ: A participant asked to clarify the part “fish (not fried)”.	The question was modified to include the following words “grilled/boiled/not fried”.
	DKQ: Many adolescents did not know what fibers and saturated fatty acids are.	No modifications made because this is related to the level of knowledge, and is an essential part of the DKQ.
2. Logical Problems	DKQ: One of the participants asked if smoking includes water pipes.	The words “cigarettes and waterpipes” were added to make it clear that both ways of smoking are included.
	DAQ/DKQ: One of the respondents asked if the word “vegetables” in question 10 (DKQ) and question 13 (DAQ) included pickles too, or just fresh vegetables like cucumbers.	In the DKQ, participants are asked if vegetables are high or low in sodium, and the word “fresh” was added to make it clearer. In the DAQ, it was left as is because both pickles and fresh vegetables are included. The purpose of this question is not related to sodium consumption, but rather to fiber intake.

**Table 3:** Demographic characteristics of the participants.

	Adolescents (N = 220)	Parents (N = 108)
Mean (SD) or n (%)		
Age, mean (SD)	16.8 (0.8)	44.3 (6.9)
Gender, n (%)		
Male	90 (40.9)	11 (19.6)
Female	129 (58.6)	45 (80.4)
Location, n (%)		
Beirut (U)	53 (24.1)	7 (6.5)
Baalbeck (R)	117 (53.2)	64 (59.3)
Rayak (R)	50 (22.7)	37 (34.3)
Type of school, n (%)		
Public	187 (85.0)	92 (85.2)
Private	33 (15.0)	16 (14.8)

U: urban; R: rural.

**Table 4:** Values for Cronbach’s alpha for the different questionnaire groups.

Scores	Value of Cronbach’s Alpha
DKQ—Adolescents	0.78
DKQ—Parents	0.89
DAQ—Adolescents, Healthy Items	0.61
DAQ—Adolescents, Unhealthy Items	0.61
DAQ—Parents, Healthy Items	0.46
DAQ—Parents, Unhealthy Items	0.61

**Table 5:** Means and differences in total knowledge and adherence scores among adolescents and their parents (maximum points: 56 for dietary knowledge (DKQ) and 37 for dietary adherence (DAQ)).

	Adolescents				Parents			
	Mean Total DKQ Knowledge Score (SD)	*p* Value	Mean Total DAQ Adherence Index (SD)	*p* Value	Mean Total DKQ Knowledge Score (SD)	*p* Value	Mean Total DAQ Adherence Index (SD)	*p* Value
Gender								
*Boys*	30.4 (7.2)	0.55	2.3 (1.8)	0.20	31.0 (10.8)	0.31	2.4 (1.7)	0.91
*Girls*	30.9 (6.8)		2.0 (1.1)		33.0 (9.6)		2.5 (2.4)	
Age/class								
*Grade 11*	29.8 (7.2)	0.07	2.5 (1.6)	0.06	33.5 (10.6)	0.20	2.0 (1.3)	0.12
*Grade 12*	31.6 (6.5)		2.0 (1.4)		30.8 (10.2)		2.8 (2.9)	
Type of school								
*Public*	30.5 (7.1)	0.20	2.1 (1.4)	0.71	32.5 (9.9)	0.77	2.4 (2.2)	0.64
*Private*	32.0 (6.1)		2.2 (2.0)		31.7 (10.8)		2.7 (2.5)	
Location								
*Urban*	33.0 (6.9)	<0.01	2.6 (1.9)	0.07	38.3 (12.1)	0.11	3.8 (2.3)	0.13
*Rural*	29.9 (6.8)		2.0 (1.2)		32.0 (9.8)		2.4 (2.2)	
*Beirut (U)*	33.0 (6.9)	0.02	2.6 (1.9)	0.06	38.3 (12.1)	0.27	3.8 (2.3)	0.22
*Baalbeck (R)*	29.9 (6.9)		1.9 (1.0)		31.9 (10.0)		2.2 (2.4)	
*Rayak (R)*	30.2 (6.6)		2.2 (1.7)		32.3 (9.6)		2.6 (1.9)	

U: Urban; R: Rural.

**Table 6:** Correlations between the knowledge and adherence scores among participants.

	Total Adherence Score—Adolescents	Healthy Items—Adolescents (DAQ)	Unhealthy Items—Adolescents (DAQ)	Total Knowledge Score—Parents
Total Knowledge score—Adolescents	0.10	0.01	−0.16 *	0.36 **
Total Adherence score—Adolescents		0.30 **	−0.58 **	0.24 *
Healthy Items—Adolescents			0.33 **	0.05
Unhealthy Items—Adolescents				−0.33 **

* *p* < 0.05; ** *p* < 0.01.

## Supplementary Materials

The following are available online at https://www.mdpi.com/1660-4601/17/1/147/s1, S1: Dietary Knowledge questionnaire in English; S2: Dietary Knowledge Questionnaire in Arabic; S3: DKQ for parents in English; S4: DKQ for parents in Arabic; S5: Dietary Adherence Questionnaire in English; S6: Dietary Adherence Questionnaire in Arabic; S7: DAQ for parents in English; S8: DAQ for parents in Arabic.

## Appendix A

**Table A1:** Dietary Knowledge Questionnaire (DKQ) structure.

Parts	Subject	Purpose	Number of Items/maximum Points ^a^
1	Knowledge of dietary recommendations	To determine whether participants are familiar with the current dietary recommendations	6
2	Knowledge of nutrient sources	To determine whether participants know if the indicated food items are good or bad sources of the nutrient in question	37
3	Knowledge of common misconceptions in nutrition	To determine whether participants are aware of some misconceptions related to nutrition	4
4	Using knowledge in nutrition to make dietary choices	To determine whether participants are able to choose the healthiest option when given different food choices	4
5	Knowledge of associations between nutrition and diseases	To determine whether participants are aware about the correlation between eating habits/smoking and health	5
		Total	56

^a^ Each correct item corresponds to 1 point on the DKQ.

## Appendix B

**Table A2:** Structure of the dietary adherence questionnaire.

Parts	Subject	Number of Items
1	Food choices	21
2	Eating habits	4
3	PA and screen-viewing time	4
4	Smoking	1

PA: physical activity.

**Table A3:** Scoring system for total adherence score calculation.

Questions	Options	Points
1–21	No, I did not eat any of the foods listed above	0
	Yes, I ate it once	1
	Yes, I ate it twice	2
	Yes, I ate it three times or more	3
22	Yes	2
	No	0
23 and 25	No, I did not have any meal	0
	Yes, I had one meal	1
	Yes, I had two meals	2
	Yes, I had 3 meals	3
26 and 27	Yes	1
	No	0
28	0 h	0
	1 h	1
	2–4 h	2
	≥5 h	3
30	Yes	2
	No/stopped smoking	1

**Table A4:** Total adherence score components for the parents.

Items	Questions	Maximum Points
**Healthy Items**	1—Yesterday, did you eat meat (like chopped meat in stews), chicken breast (grilled/boiled/not fried), or fish (grilled/boiled/not fried)? 4—Yesterday, did you eat any of these foods? Labneh, shanklish, kareesha 5—Yesterday, did you eat any of these cheeses: Mozzarella, feta, akawi, baladiye, khadra? 8—Yesterday, did you drink skimmed milk (reduced or 0% fat) or skimmed laban? 10—Yesterday, did you eat whole bread, oat bread, brown bread, tortillas? 12—Yesterday, did you eat beans like lentils, white beans, fava beans (do not count green beans)? 13—Yesterday, did you eat any vegetables (do not count potatoes)? 14—Yesterday, did you eat fresh fruits? Do not count fruit juice and dried fruits. 22—Yesterday, did you have breakfast? 23—Yesterday, how many meals did you eat (meals include breakfast, lunch and dinner)? 26—Do you practice physical activity regularly? 27—How many hours per week do you exercise?	33
**Unhealthy Items**	2—Yesterday, did you eat fried chicken, chicken nuggets, fried fish, fried meat, hot dogs, sausage, mortadella, or ham? 3—Yesterday, did you eat chocolate cream or potato chips? 6—Yesterday, did you eat any of these foods: Cheddar cheese, gruyere, edam, goat cheese, gouda, parmesan, Roquefort, kashkawan, cream cheese, kishk? 7—Yesterday, did you drink whole milk or whole laban? 11—Yesterday, did you eat French fries (fried potatoes)? 17—Yesterday, did you drink soft drinks (Pepsi, 7up, Miranda, Sprite, Coca Cola, Fanta …)? 19—Yesterday, did you drink energy drinks (Red Bull, AMP, …)? 20—Yesterday, did you eat any sweets such as sweet rolls, cookies, cakes, pies, brownies, cheesecake, doughnuts? 21—Yesterday, did you eat any Arabic sweets (namoura, knefeh, halewet el jibn, znood el sitt …)? 25—Yesterday, how many times did you eat food from outside of your house? (restaurants, fast food restaurants, pizza places, and cafeterias) 30—Do you smoke? (cigarettes and narjileh are included)	32

Remarks:

Some items were not included in the total adherence score for the following reasons:

- To improve reliability, by removing the question related to refined bread and commercial juices, our Cronbach’s alpha value increases.
- Some items are acceptable to eat by children, but are then less recommended to consume as adults, and vice versa. This explains the reason behind considering whole milk/laban as part of the unhealthy items for adults.
- To avoid controversy, diet soft drinks were removed from both scores for adults, as it depends on the overall health of each individual separately.

## Appendix C

**Table A5:** Responses to the dietary knowledge questionnaire by adolescents.

	Number of Answers	Percent
Recommended number of fruit and vegetable servings per day		
False/Do not know	198	90.0
Correct answers	22	10.0
Type of fat to cut down on		
False/Do not know	117	53.2
Correct answers	103	46.8
Recommended type of dairy products		
False/Do not know	160	72.7
Correct answers	60	27.3
Recommended daily water intake		
False/Do not know	105	47.7
Correct answers	115	52.3
Recommended duration of daily physical activity		
False/Do not know	93	42.3
Correct answers	127	57.7
Recommended intake of energy drinks per day		
False/Don’t know	102	46.4
Correct answers	118	53.6
Do you think these are high or low in added sugar?		
Apples		
False/Do not know	53	24.1
Correct answers	167	75.9
Ice cream		
False/Do not know	47	21.4
Correct answers	173	78.6
Commercial juices		
False/Do not know	12	5.5
Correct answers	208	94.5
Soft drinks		
False/Do not know	26	11.8
Correct answers	194	88.2
Grapes		
False/Do not know	66	30.0
Correct answers	154	70.0
Do you think these foods are high or low in fat?		
Pasta		
False/Do not know	83	37.7
Correct answers	137	62.3
Beans		
False/Do not know	60	27.3
Correct answers	160	72.7
Honey		
False/Do not know	152	69.1
Correct answers	68	30.9
Nuts		
False/Do not know	88	40.0
Correct answers	132	60.0
Bread		
False/Do not know	128	58.2
Correct answers	92	41.8
Cheddar cheese		
False/Do not know	90	40.9
Correct answers	130	59.1
Chips		
False/Do not know	60	27.3
Correct answers	160	72,7
Kashkawan cheese		
False/Do not know	63	28.6
Correct answers	157	71.4
Mortadella		
False/Do not know	85	38.6
Correct answers	135	61.4
Do you think these are high or low in SFA?		
Whole milk		
False/Do not know	103	46.8
Correct answers	117	53.2
Olive oil		
False/Do not know	112	50.9
Correct answers	108	49.1
Margarine		
False/Do not know	38	17.3
Correct answers	182	82.7
Chocolate cream		
False/Do not know	70	31.8
Correct answers	150	68.2
Do you think these are high or low in salt?		
Sausage		
False/Do not know	121	55.0
Correct answers	99	45.0
Meat		
False/Do not know	83	37.7
Correct answers	137	62.3
Vegetables		
False/Do not know	24	10.9
Correct answers	196	89.1
Potato chips		
False/Do not know	23	10.5
Correct answers	197	89.5
Fries		
False/Do not know	46	20.9
Correct answers	174	79.1
Do you think these are high or low in protein?		
Chicken		
False/Do not know	45	20.5
Correct answers	175	79.5
Cheese		
False/Do not know	128	58.2
Correct answers	92	41.8
Fruits		
False/Do not know	150	68.2
Correct answers	70	31.8
Beans		
False/Do not know	39	17.7
Correct answers	181	82.3
Butter		
False/Do not know	77	35.0
Correct answers	143	65.0
Eggs		
False/Do not know	24	10.9
Correct answers	196	89.1
Do you think these are high or low in fibers?		
Eggs		
False/Do not know	122	55.5
Correct answers	98	44.5
Meat		
False/Do not know	132	60.0
Correct answers	88	40.0
Broccoli		
False/Do not know	89	40.5
Correct answers	131	59.5
Nuts		
False/Do not know	169	76.8
Correct answers	51	23.2
Fish		
False/Do not know	155	70.5
Correct answers	65	29.5
Beans		
False/Do not know	117	53.2
Correct answers	103	46.8
Rice		
False/Do not know	153	69.5
Correct answers	67	30.5
Bulgur		
False/Do not know	142	64.5
Correct answers	78	35.5
True/False (T/F): Some foods contain a lot of fat, but no cholesterol		
False/Do not know	165	75.0
Correct answers	55	25.0
T/F: Brown sugar is a healthy alternative to white sugar		
False/Do not know	180	81.8
Correct answers	40	18.2
T/F: There is more protein in a glass of whole milk than in a glass of skimmed milk		
False/Do not know	173	78.6
Correct answers	47	21.4
A type of oil which contains mostly unsaturated fatty acids is:		
False/Do not know	160	72.7
Correct answers	60	27.3
Which of these breads contain the most vitamins and minerals?		
False/Do not know	133	60.5
Correct answers	87	39.5
T/F: There is more Calcium in a glass of whole milk than a glass of skimmed milk		
False/Do not know	195	88.6
Correct answers	25	11.4
If a person wanted to reduce the amount of fat in their diet, which would be the best choice?		
False/Do not know	99	45.0
Correct answers	121	55.0
Which cheese would be the best choice as a lower fat option?		
False/Do not know	173	78.6
Correct answers	47	21.4
What do you think will help prevent heart diseases?		
Increasing fiber intake		
False/Do not know	96	43.6
Correct answers	124	56.4
Decreasing salt intake		
False/Do not know	42	19.1
Correct answers	178	80.9
Increasing saturated fatty acid intake		
False/Do not know	63	28.6
Correct answers	157	71.4
Are you aware that many chronic diseases (such as diabetes, heart diseases and certain types of cancer) are related to a low intake of fruits and vegetables?		
False/Do not know	119	54.1
Correct answers	101	45.9
Are you aware that smoking causes many chronic diseases (such as lung cancer and heart diseases)?		
False/Do not know	21	9.5
Correct answers	199	90.5

**Table A6:** Responses to the dietary adherence questionnaire by adolescents.

	Frequency	Percent
Yesterday, did you eat meat (like chopped meat in stews), chicken breast (not fried), or fish (not fried)? *		
None	109	49.5
Once	95	43.2
Twice	11	5.0
≥Three times	3	1.4
Yesterday, did you eat fried chicken, chicken nuggets, fried fish, fried meat, hot dogs, sausage, mortadella, or ham? **		
None	160	72.7
Once	51	23.2
Twice	4	1.8
≥Three times	2	0.9
Yesterday, did you eat chocolate cream or potato chips? **		
None	62	28.2
Once	121	55.0
Twice	21	9.5
≥Three times	14	6.4
Yesterday, did you eat any of these foods? Labneh, shanklish, kareesha? *		
None	109	49.5
Once	94	42.7
Twice	12	5.5
≥Three times	1	0.5
Yesterday, did you eat any of these cheeses? Mozzarella, feta, akawi, baladiye, khadra? *		
None	139	63.2
Once	69	31.4
Twice	7	3.2
≥Three times	2	0.9
Yesterday, did you eat any of these foods? Cheddar cheese, gruyere, edam, goat cheese, gouda, parmesan, Roquefort, kashkawan, cream cheese, kishk **		
None	180	81.8
Once	35	15.9
Twice	1	0.5
≥Three times	2	0.9
Yesterday, did you drink milk or laban? *		
None	144	65.5
Once	71	32.3
Twice	2	0.9
≥Three times	1	0.5
Yesterday, did you drink skimmed milk (reduced or 0% fat) or skimmed laban? *		
None	184	83.6
Once	31	14.1
Twice	2	0.9
≥Three times	1	0.5
Yesterday, did you eat white Arabic bread, kaak, franjeh bread? **		
None	49	22.3
Once	119	54.1
Twice	38	17.3
≥Three times	12	5.5
Yesterday, did you eat whole bread, oat bread, brown bread, tortillas? *		
None	142	64.5
Once	59	26.8
Twice	9	4.1
≥Three times	7	3.2
Yesterday, did you eat French fries (fried potatoes)? **		
None	127	57.7
Once	81	36.8
Twice	8	3.6
≥Three times	2	0.9
Yesterday, did you eat beans like lentils, white beans, fava beans (do not count green beans)? *		
None	172	78.2
Once	44	20.0
Twice	1	0.5
≥Three times	1	0.5
Yesterday, did you eat any vegetables (do not count potatoes)? *		
None	60	27.3
Once	131	59.5
Twice	19	8.6
≥Three times	8	3.6
Yesterday, did you eat fresh fruits? Do not count fruit juice and dried fruits *		
None	81	36.8
Once	110	50.0
Twice	18	8.2
≥Three times	9	4.1
Yesterday, did you drink natural fruit juice?		
None	153	69.5
Once	61	27.7
Twice	3	1.4
≥Three times	1	0.5
Yesterday, did you drink commercial fruit juice? **		
None	150	68.2
Once	59	26.8
Twice	4	1.8
≥Three times	3	1.4
Yesterday, did you drink soft drinks (Pepsi, 7up, Miranda, Sprite, Coca Cola, Fanta …)? **		
None	98	44.5
Once	85	38.6
Twice	26	11.8
≥Three times	9	4.1
Yesterday, did you drink diet soft drinks (Pepsi diet, 7up diet, Coca Cola diet, …)? **		
None	207	94.1
Once	8	3.6
Twice	2	0.9
≥Three times	0	0
Yesterday, did you drink energy drinks (Red Bull, AMP, …)? **		
None	198	90.0
Once	15	6.8
Twice	3	1.4
≥Three times	1	0.5
Yesterday, did you eat any sweets such as sweet rolls, cookies, cakes, pies, brownies, cheesecake, doughnuts? **		
None	156	70.9
Once	50	22.7
Twice	10	4.5
≥Three times	0	0
Yesterday, did you eat any Arabic sweets (namoura, knefeh, halewet el jibn, znood el sitt …)? **		
None	169	76.8
Once	47	21.4
Twice	2	0.9
≥Three times	0	0
Yesterday, did you have breakfast? *		
No	49	22.3
Yes	168	76.4
Yesterday, how many meals did you eat (meals include breakfast, lunch, and dinner)? *		
None	1	0.5
One meal	36	16.4
2 meals	100	45.5
3 meals	81	36.8
Yesterday, did you have a snack? A snack is a food or drink (except for water) that you eat or drink between meals.		
None	63	28.6
1 snack	93	42.3
2 snacks	42	19.1
≥3 snacks	17	7.7
Yesterday, how many times did you eat food from outside of your house? (restaurants, fast food restaurants, pizza places and cafeterias) **		
None	169	76.8
One meal	36	16.4
2 meals	10	4.5
≥3 meals	3	1.4
Do you participate in physical education sessions at school? *		
No	60	27.3
Yes	158	71.8
Do you participate in any sports activity (other than physical education session at schools)? *		
No	99	45.0
Yes	117	53.2
How many hours per week do you exercise? *		
0 h	36	16.4
1 h	68	30.9
2–4 h	66	30.0
≥5 h	48	21.8
How many hours per day do you usually spend playing video games, watching TV, using tablets and chatting on the phone?		
0 h	9	4.1
1 h	39	17.7
2 h	36	16.4
3 h	31	14.1
4 h	36	16.4
5 h	20	9.1
≥6 h	48	21.8
Do you smoke? (cigarettes and narjileh are included) **		
No	149	67.7
Yes	65	29.5
Stopped smoking	4	1.8

*: Questions included in healthy items score; **: Questions included in unhealthy items score.

## Appendix D

**Table A7:** Total knowledge score differences between the three locations (Beirut, Baalbeck and Rayak).

Total Knowledge Score	Mean Difference (SE)
Beirut vs. Baalbeck	3.14 * (1.14)
Beirut vs. Rayak	2.82 (1.35)
Baalbeck vs. Rayak	−0.32 (1.16)

* *p* < 0.05.

**Table A8:** Total adherence score differences between the three locations (Beirut, Baalbeck and Rayak).

Total Adherence Score	Mean Difference (SE)
Beirut vs. Baalbeck	0.61 * (0.25)
Beirut vs. Rayak	0.40 (0.30)
Baalbeck vs. Rayak	−0.20 (0.26)

* *p* < 0.05.

## Appendix E

**Table A9:** Responses to the dietary knowledge questionnaire by parents/caregivers.

	Number of Answers	Percent
Recommended number of fruit and vegetable servings per day		
False/Do not know	98	90.7
Correct answers	10	9.3
Type of fat to cut down		
False/Do not know	28	25.9
Correct answers	80	74.1
Recommended type of dairy products		
False/Do not know	74	68.5
Correct answers	34	31.5
Recommended daily water intake		
False/Do not know	58	53.7
Correct answers	50	46.3
Recommended duration of daily physical activity		
False/Do not know	41	38.0
Correct answers	67	62.0
Recommended intake of energy drinks per day		
False/Do not know	35	32.4
Correct answers	73	67.6
Do you think these are high or low in added sugar?		
Apples		
False/Do not know	33	30.6
Correct answers	75	69.4
Ice cream		
False/Do not know	22	20.4
Correct answers	86	79.6
Commercial juices		
False/Do not know	16	14.8
Correct answers	92	85.2
Soft drinks		
False/Do not know	15	13.9
Correct answers	93	86.1
Grapes		
False/Do not know	40	37.0
Correct answers	68	63.0
Do you think these foods are high or low in fat?		
Pasta		
False/Do not know	39	36.1
Correct answers	69	63.9
Beans		
False/Do not know	26	24.1
Correct answers	82	75.9
Honey		
False/Do not know	53	49.1
Correct answers	55	50.9
Nuts		
False/Do not know	31	28.7
Correct answers	77	71.3
Bread		
False/Do not know	65	60.2
Correct answers	43	39.8
Cheddar cheese		
False/Do not know	24	22.2
Correct answers	84	77.8
Chips		
False/Do not know	32	29.6
Correct answers	76	70.4
Kashkawan cheese		
False/Do not know	22	20.4
Correct answers	86	79.6
Mortadella		
False/Do not know	36	33.3
Correct answers	72	66.7
Do you think these are high or low in saturated fatty acids?		
Whole milk		
False/Do not know	37	34.9
Correct answers	69	65.1
Olive oil		
False/Do not know	43	40.6
Correct answers	63	59.4
Margarine		
False/Do not know	13	12.3
Correct answers	93	87.7
Chocolate cream		
False/Do not know	27	25.5
Correct answers	79	74.5
Do you think these are high or low in salt?		
Sausage		
False/Do not know	39	36.8
Correct answers	67	63.2
Meat		
False/Do not know	49	46.2
Correct answers	57	53.8
Vegetables		
False/Do not know	15	14.2
Correct answers	91	85.8
Potato chips		
False/Do not know	9	8.6
Correct answers	96	91.4
Fries		
False/Do not know	25	23.8
Correct answers	80	76.2
Do you think these are high or low in protein?		
Chicken		
False/Do not know	25	23.8
Correct answers	80	76.2
Cheese		
False/Do not know	59	56.2
Correct answers	46	43.8
Fruits		
False/Do not know	56	53.8
Correct answers	48	46.2
Beans		
False/Do not know	32	30.8
Correct answers	72	69.2
Butter		
False/Do not know	51	49.0
Correct answers	53	51.0
Eggs		
False/Do not know	12	11.5
Correct answers	92	88.5
Do you think these are high or low in fiber?		
Eggs		
False/Do not know	44	42.3
Correct answers	60	57.7
Meat		
False/Do not know	57	54.8
Correct answers	47	45.2
Broccoli		
False/Do not know	31	30.1
Correct answers	72	69.9
Nuts		
False/Do not know	87	83.7
Correct answers	17	16.3
Fish		
False/Do not know	60	57.7
Correct answers	44	42.3
Beans		
False/Do not know	46	44.2
Correct answers	58	55.8
Rice		
False/Do not know	52	50.0
Correct answers	52	50.0
Bulgur		
False/Do not know	61	58.7
Correct answers	43	41.3
T/F: Some foods contain a lot of fat but no cholesterol		
False/Do not know	71	68.3
Correct answers	33	31.7
T/F: Brown sugar is a healthy alternative to white sugar		
False/Do not know	88	84.6
Correct answers	16	15.4
T/F: There is more protein in a glass of whole milk than in a glass of skimmed milk		
False/Do not know	79	76.0
Correct answers	25	24.0
A type of oil which contains mostly unsaturated fatty acids is:		
False/Do not know	62	59.6
Correct	42	40.4
Which of the indicated breads contain the most vitamins and minerals		
False/Do not know	46	44.2
Correct answers	58	55.8
T/F: There is more Calcium in a glass of whole milk than a glass of skimmed milk		
False/Do not know	72	69.2
Correct answers	32	30.8
If a person wanted to reduce the amount of fat in their diet, which type of meat would be the best choice?		
False/Do not know	35	33.7
Correct answers	69	66.3
Which cheese would be the best choice as a lower fat option?		
False/Do not know	86	82.7
Correct answers	18	17.3
What do you think will help prevent heart diseases?		
Increasing fiber intake		
False/Do not know	37	35.6
Correct answers	67	64.4
Decreasing salt intake		
False/Do not know	25	24.0
Correct answers	79	76.0
Increasing saturated fatty acid intake		
False/Do not know	33	31.7
Correct answers	71	68.3
Are you aware that many chronic diseases (such as diabetes, heart diseases and certain types of cancer) are related to a low intake of fruits and vegetables?		
False/Do not know	54	51.9
Correct answers	50	48.1
Are you aware that smoking causes many chronic diseases (such as lung cancer and heart diseases)?		
False/Do not know	13	12.5
Correct answers	91	87.5

**Table A10:** Answers to the dietary adherence questionnaire per question by parents.

	Number of Answers	Percent
Yesterday, did you eat meat (like chopped meat in stews), chicken breast (not fried), or fish (not fried)? *		
None	44	44.0
Once	53	53.0
Twice	3	3.0
≥Three times	0	0
Yesterday, did you eat fried chicken, chicken nuggets, fried fish, fried meat, hot dogs, sausage, mortadella, or ham? **		
None	74	74.0
Once	24	24.0
Twice	2	2.0
≥Three times	0	0
Yesterday, did you eat chocolate cream or potato chips? **		
None	40	40.0
Once	51	51.0
Twice	8	8.0
≥Three times	1	1.0
Yesterday, did you eat any of these foods? Labneh, shanklish, kareesha? *		
None	42	42.0
Once	55	55.0
Twice	2	2.0
≥Three times	1	1.0
Yesterday, did you eat any of these cheeses? Mozzarella, feta, akawi, baladiye, khadra? *		
None	72	72.0
Once	26	26.0
Twice	2	2.0
≥Three times	0	0
Yesterday, did you eat any of these foods? Cheddar cheese, gruyere, edam, goat cheese, gouda, parmesan, Roquefort, kashkawan, cream cheese, kishk **		
None	77	77.0
Once	21	21.0
Twice	1	1.0
≥Three times	1	1.0
Yesterday, did you drink milk or laban? **		
None	44	44.0
Once	51	51.0
Twice	3	3.0
≥Three times	2	2.0
Yesterday, did you drink skimmed milk (reduced or 0% fat) or skimmed laban? *		
None	80	80.0
Once	16	16.0
Twice	3	3.0
≥Three times	1	1.0
Yesterday, did you eat white Arabic bread, kaak, franjeh bread?		
None	17	17.2
Once	53	53.5
Twice	23	23.2
≥Three times	6	6.1
Yesterday, did you eat whole bread, oat bread, brown bread, tortillas? *		
None	66	66.7
Once	23	23.2
Twice	7	7.1
≥Three times	3	3.0
Yesterday, did you eat French fries (fried potatoes)? **		
None	46	46.0
Once	47	47.0
Twice	3	3.0
≥Three times	4	4.0
Yesterday, did you eat beans (do not count green beans) like lentils, white beans, fava beans? *		
None	72	72.0
Once	22	22.0
Twice	6	6.0
≥Three times	0	0
Yesterday, did you eat any vegetables (do not count potatoes)? *		
None	19	19.0
Once	67	67.0
Twice	12	12.0
≥Three times	2	2.0
Yesterday, did you eat fresh fruits? Do not count fruit juice and dried fruits? *		
None	26	25.7
Once	52	51.5
Twice	20	19.8
≥Three times	3	3.0
Yesterday, did you drink natural fruit juice?		
None	68	67.3
Once	29	28.7
Twice	4	4.0
≥Three times	0	0
Yesterday, did you drink commercial fruit juice?		
None	72	71.3
Once	24	23.8
Twice	1	1.0
≥Three times	4	4.0
Yesterday, did you drink soft drinks (Pepsi, 7up, Miranda, Sprite, Coca Cola, Fanta …)? **		
None	55	54.5
Once	37	36.6
Twice	8	7.9
≥Three times	1	1.0
Yesterday, did you drink diet soft drinks (Pepsi diet, 7up diet, Coca Cola diet, …)?		
None	90	90.0
Once	7	7.0
Twice	2	2.0
≥Three times	1	1.0
Yesterday, did you drink energy drinks (Red Bull, AMP, …)? **		
None	95	93.1
Once	4	3.9
Twice	2	2.0
≥Three times	1	1.0
Yesterday, did you eat any sweets such as sweet rolls, cookies, cakes, pies, brownies, cheesecake, doughnuts? **		
None	72	70.6
Once	24	23.5
Twice	4	3.9
≥Three times	2	2.0
Yesterday, did you eat any Arabic sweets (namoura, knefeh, halewet el jibn, znood el sitt …)? **		
None	75	72.8
Once	26	25.2
Twice	1	1.0
≥Three times	1	1.0
Yesterday, did you have breakfast? *		
No	14	13.9
Yes	87	86.1
Yesterday, how many meals did you eat (meals include breakfast, lunch and dinner)? *		
None	3	3.0
One meal	10	9.9
2 meals	28	27.7
3 meals	60	59.4
Yesterday, did you have a snack? A snack is a food or drink (except for water) that you eat or drink between meals.		
None	37	36.6
1 snack	44	43.6
2 snacks	15	14.9
≥3 snacks	5	5.0
Yesterday, how many times did you eat food from outside of your house? (restaurants, fast food restaurants, pizza places and cafeterias) **		
None	87	87.9
One meal	11	11.1
2 meals	1	1.0
≥3 meals	0	0
Do you practice physical activity on a regular basis? And for how many hours per week? *		
No	51	50.0
Yes	51	50.0
0 h	26	26.0
1 h	30	30.0
2–4 h	24	24.0
≥5 h	20	20.0
How many hours per day do you usually spend playing video games, watching TV, using tablets and chatting on the phone?		
0 h	3	3.0
1 h	22	21.8
2 h	25	24.8
3 h	21	20.8
4 h	14	13.9
5 h	6	5.9
≥6 h	10	9.9
Do you smoke? (cigarettes and narjileh are included) **		
No	60	60.0
Yes	35	35.0
Stopped smoking	5	5.0

*: Questions included in healthy items score; **: Questions included in unhealthy items score.

## Appendix F

**Table A11:** Pearson’s correlations between the DAQ items and the corresponding items based on the 24 h recall.

Items	Pearson Correlation
Lean meat	0.29 ***
High fat meat	0.30 ***
Unhealthy snacks (potato chips and chocolate cream)	0.48 ***
Low fat dairy	0.54 ***
Medium fat dairy	0.30 ***
High fat dairy	0.32 ***
Whole milk	0.42 ***
Skimmed milk	0.14 ^†^
Refined bread	0.27 ***
Whole bread	0.15 *
French fries	0.53 ***
Beans	0.45 ***
Vegetables	0.38 ***
Fruits	0.49 ***
Natural juices	0.52 ***
Commercial fruit juices	0.45 ***
Soft drinks	0.56 ***
Diet soft drinks	0.13 ^†^
Energy drinks	0.51 ***
Sweets	0.30 ***
Arabic sweets	0.33 ***
Breakfast	0.66 ***
Number of meals	0.61 ***
Number of snacks	0.20 **
Meals eaten outside	0.40 ***

*** *p* < 0.001; ** *p* < 0.01; * *p* < 0.05; ^†^ *p* < 0.10.
