# Supplementary material for: Development of Dietary Knowledge and Adherence Questionnaires for Lebanese Adolescents and Their Parents
Source: Int J Environ Res Public Health. 2019 Dec 24;17(1):147. doi: 10.3390/ijerph17010147 (PMC6982025; doi:10.3390/ijerph17010147)
Supplement: Supplementary file 1 [file ijerph-17-00147-s001.zip › Supplementary material - S1 - DKQ En.pdf]

Name:

Dietary Knowledge Questionnaire

- **ID:**
- **School's name:**
- **Age:**
- **Birth month:**
  - January
  - April
  - July
  - October
  - February
  - May
  - August
  - November
  - March
  - June
  - September
  - December
- **Birth year:**
  - 1999
  - 2000
  - 2001
  - Other: ______
- **Grade:**
  - Grade 10
  - Grade 11
  - Grade 12
- **Boy/girl:**
  - Boy
  - Girl
- **Address city (city/village):**

* You are invited to participate in a scientific research conducted by Maastricht University in the Netherlands for the academic year 2016-2017.
* The purpose of this study is to assess the dietary behavior of Lebanese adolescents
* You will be asked to answer 23 questions. Completing the questionnaire will take approximately 15 minutes.
* You are free to withdraw at any time. Participation is voluntary.
* All the collected data will be anonymous, and answers will not be revealed.
* Feel free to ask any additional question.
* By filling this questionnaire, you agree to participate in this project.

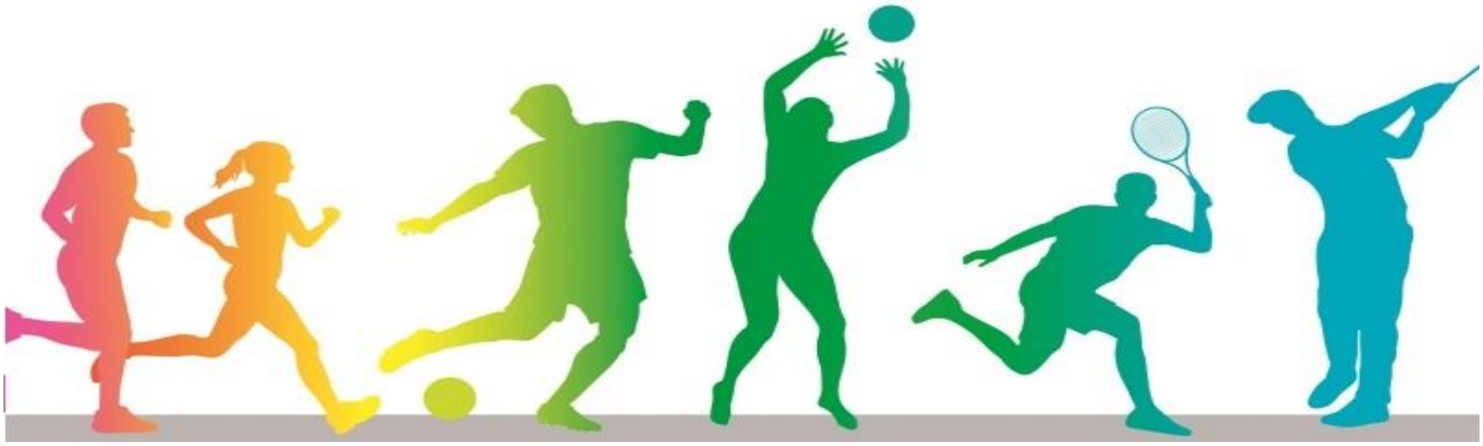

sahtak bi Sahnak

1. **How many servings of fruits and vegetables is it recommended to eat per day?**
    - ○ 0-1 ○ 2-4 ○ 5 or more ○ Not sure

2. **Which type of fat is most important for people to cut down on?**
    - ○ Unsaturated fatty acids ○ Saturated fatty acids ○ Not sure

3. **What version of dairy foods should be eaten more than the others?**
    - ○ Full fat ○ Lower fat ○ Mixture of both full and lower fat ○ Neither, dairy products should be cut off ○ Not sure

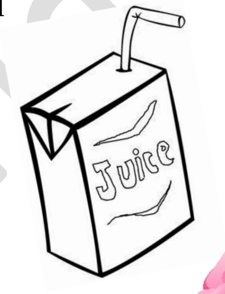

4. **How many cups of water is it recommended to drink per day?**
    - ○ 1-3 ○ 4-7 ○ 8 ○ Not sure

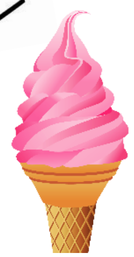

5. **How much time should you spend on daily work-out?**
    - ○ 10-15 minutes ○ 15-20 minutes ○ 30 minutes or more ○ Not sure

6. **How many cups of energy drinks are you allowed to drink per day?**
    - ○ 0 ○ 1-2 ○ 3-4 ○ Not sure

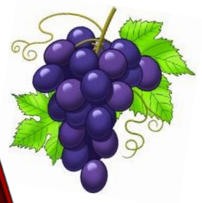

7. **Do you think these are high or low in added sugar?**

Apples	○ High	○ Low	○ Not sure
Ice cream	○ High	○ Low	○ Not sure
Commercial fruit juice	○ High	○ Low	○ Not sure
Soft drinks	○ High	○ Low	○ Not sure
Grapes	○ High	○ Low	○ Not sure

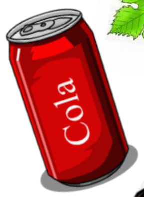

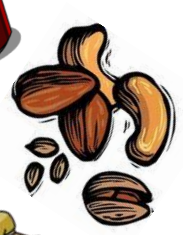

8. **Do you think these foods are high or low in fat?**

Pasta (without sauces)	○ High	○ Low	○ Not sure
Beans	○ High	○ Low	○ Not sure
Honey	○ High	○ Low	○ Not sure
Nuts	○ High	○ Low	○ Not sure
Bread	○ High	○ Low	○ Not sure
Cheddar cheese	○ High	○ Low	○ Not sure
Chips	○ High	○ Low	○ Not sure
Kashkawan	○ High	○ Low	○ Not sure
Mortadella	○ High	○ Low	○ Not sure

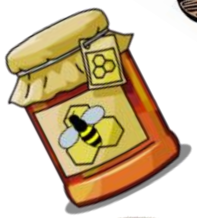

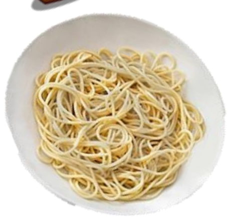

sahtak bi sahnak

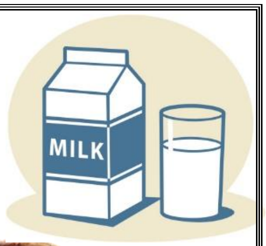

9. Do you think these are high or low in saturated fatty acids?

Whole milk	○ High	○ Low	○ Not sure
Olive oil	○ High	○ Low	○ Not sure
Margarine	○ High	○ Low	○ Not sure
Chocolate paste	○ High	○ Low	○ Not sure

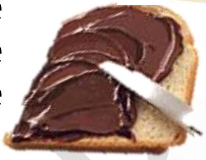

10. Do you think these are high or low in salt?

Sausages	○ High	○ Low	○ Not sure
Meat	○ High	○ Low	○ Not sure
Fresh vegetables	○ High	○ Low	○ Not sure
Chips	○ High	○ Low	○ Not sure
French fries (commercial)	○ High	○ Low	○ Not sure

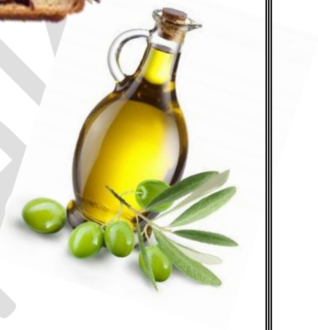

11. Do you think these are high or low in protein?

Chicken	○ High	○ Low	○ Not sure
Cheese	○ High	○ Low	○ Not sure
Fruits	○ High	○ Low	○ Not sure
Beans	○ High	○ Low	○ Not sure
Butter	○ High	○ Low	○ Not sure
Eggs	○ High	○ Low	○ Not sure

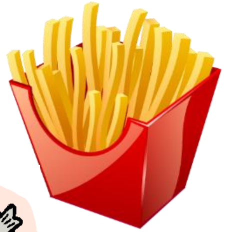

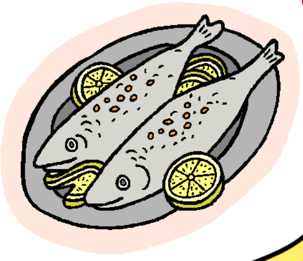

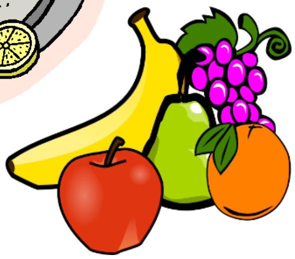

12. Do you think these are high or low in fiber?

Eggs	○ High	○ Low	○ Not sure
Red meat	○ High	○ Low	○ Not sure
Broccoli	○ High	○ Low	○ Not sure
Nuts	○ High	○ Low	○ Not sure
Fish	○ High	○ Low	○ Not sure
Beans	○ High	○ Low	○ Not sure
Rice	○ High	○ Low	○ Not sure
Bulgur	○ High	○ Low	○ Not sure

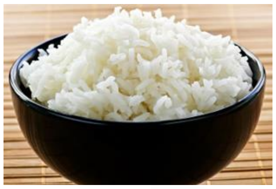

13. Some foods contain a lot of fat, but no cholesterol:

○ True ○ False ○ Not sure

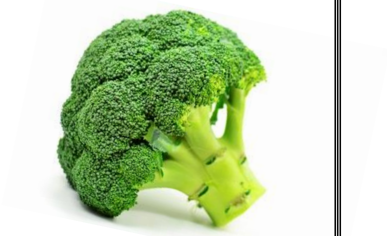

14. Brown sugar is a healthy alternative to white sugar:

○ True ○ False ○ Not sure

15. There is more protein in a glass of whole milk than in a glass of skimmed milk:

○ True ○ False ○ Not sure

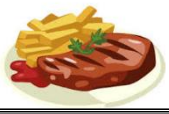
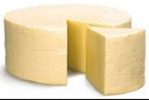
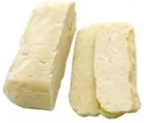
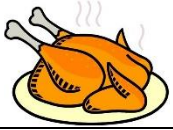
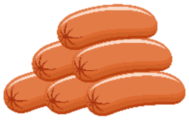

16. A type of oil which contains mostly unsaturated fatty acids is (tick one answer):

- ○ Olive oil ○ Butter ○ Palm oil ○ Not sure

17. Which of these breads contain the most vitamins and minerals (tick one answer)?

- ○ White bread ○ Whole grain bread ○ Not sure

18. There is more Calcium in a glass of whole milk than a glass of skimmed milk:

- ○ True ○ False ○ Not sure

19. If a person wanted to reduce the amount of fat in their diet, which would be the best choice?

- ○ Grilled steak ○ Grilled chicken breast ○ Sausages
- ○ Grilled lamb meat ○ Not sure

20. Which cheese would be the best choice as a lower fat option?

- ○ Kashkawan ○ Akkawi ○ Halloum ○ Cream cheese
- ○ Not sure

21. What do you think will help prevent heart diseases?

Eating more fibers	○ Yes	○ No	○ Not sure
Eating less salt	○ Yes	○ No	○ Not sure
Eating more saturated fatty acids	○ Yes	○ No	○ Not sure

22. Increased risks of chronic diseases (such as diabetes, heart diseases and certain types of cancer) are related to a low intake of fruits and vegetables:

- ○ True ○ False ○ Not sure

23. Smoking causes many chronic diseases (such as lung cancer and heart diseases):

- ○ True ○ False ○ Not sure

Thank you very much for your time.

If there are any comments you would like to make about this questionnaire, please do so below, they would be very welcome.

____

____

Adopted from: Parmenter and Wardle, European Journal of Clinical Nutrition, 53, 298-308, 1999.

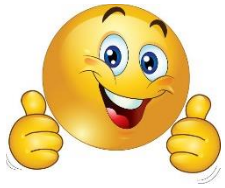

Sahtak bi Sahnak
