# Supplementary material for: Development of Dietary Knowledge and Adherence Questionnaires for Lebanese Adolescents and Their Parents
Source: Int J Environ Res Public Health. 2019 Dec 24;17(1):147. doi: 10.3390/ijerph17010147 (PMC6982025; doi:10.3390/ijerph17010147)
Supplement: Supplementary file 1 [file ijerph-17-00147-s001.zip › Supplementary material - S2 - DKQ Ar.pdf]

الاسم:

Dietary Knowledge Questionnaire

- **الرقم:**________________________
- **المدرسة:** _____________________
- **العمر:** ________
- **شهر الولادة:**
  - كانون الثاني ○ شباط ○ اذار ○ نيسان
  - ○ ايار ○ حزيران ○ تموز ○ اب
  - ○ ايلول ○ تشرين الاول ○ تشرين الثاني ○ كانون الاول
- **سنة الولادة:**
  - ○ 1999 ○ 2000 ○ 2001 ○ غير: ____
- **الصف:**
  - ○ الاول ثانوي ○ الثاني ثانوي ○ الثالث ثانوي
- **فتى/فتاة:**
  - ○ فتاة ○ فتى
- **العنوان (المدينة/القرية):** ___________

- إنك مدعو(ة) للمشاركة في بحث علمي تجريه جامعة ماسترشت في هولندا خلال العام الدراسي 2016-2017.
- تهدف هذه الدراسة إلى تقييم السلوك الغذائي للمراهقين اللبنانيين.
- سيطلب منكم الإجابة على 23 سؤال حول المعرفة الغذائية، وذلك لمدة 15 دقيقة.
- يمكنك التوقف عن المشاركة في هذا البحث خلال اي وقت. إن المشاركة في هذا المشروع هي طوعية.
- ستكون جميع الإستبيانات مجهولة الهوية، وبالتالي لن يتم الأفصاح عن اية اجوبة.
- للمزيد من المعلومات، لا تتردد بطرح الأسئلة.
- من خلال تعبئة هذا الإستبيان، إنك توافق على المشاركة في هذا المشروع.

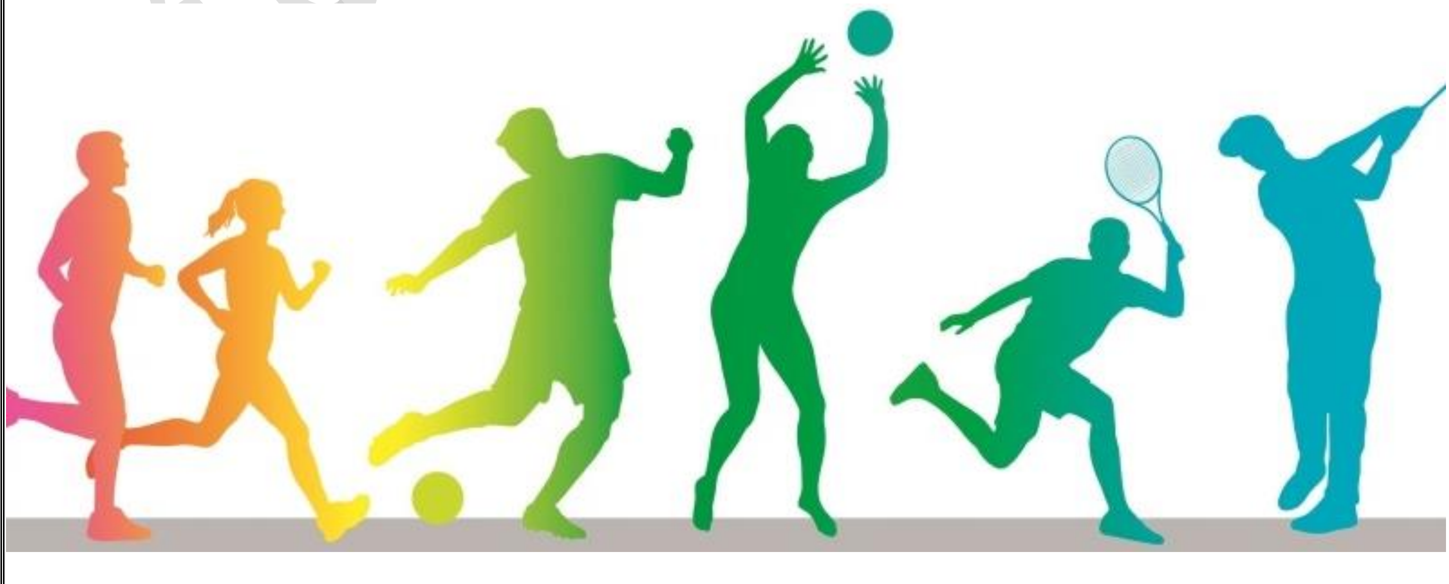

1. **كم حصة من الخضار والفاكهة ينصح بتناولها خلال النهار؟**
   - ○ 1-0
   - ○ 4-2
   - ○ 5 أو أكثر
   - ○ لست متأكد(ة)

2. **ما نوع الدسم الذي يجب تجنبه؟**
   - ○ الدهون الغير مشبعة
   - ○ الدهون المشبعة
   - ○ لست متأكد(ة)

3. **أي من مشتقات الحليب التالية يجب تناولها أكثر من غيرها؟**
   - ○ مشتقات الحليب الكاملة الدسم
   - ○ مشتقات الحليب القليلة الدسم
   - ○ علينا تناول مشتقات الحليب الكاملة الدسم والقليلة الدسم
   - ○ علينا تجنب تناول مشتقات الحليب
   - ○ لست متأكد(ة)

4. **كم كوب من الماء ينصح بالشرب خلال النهار؟**
   - ○ 3-1
   - ○ 7-4
   - ○ 8
   - ○ لست متأكد(ة)

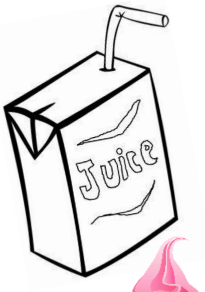

5. **لكم من الوقت ينصح بممارسة الرياضة يومياً؟**
   - ○ 10-15دقائق
   - ○ 20-15 دقيقة
   - ○ 30 دقيقة أو أكثر
   - ○ لست متأكد(ة)

6. **كم كوب من مشروب الطاقة يسمح بالشرب خلال النهار؟**
   - ○ 0
   - ○ 2-1
   - ○ 4-3
   - ○ لست متأكد(ة)

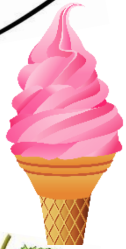

7. **برأيك، هل تحتوي المأكولات التالية على الكثير أو القليل من السكر المضاف؟**

التفاح	○ ألكثير	○ ألقليل	○ لست متأكد(ة)
المثلجات	○ ألكثير	○ ألقليل	○ لست متأكد(ة)
عصير الفاكهة المصنع	○ ألكثير	○ ألقليل	○ لست متأكد(ة)
المشروبات الغازية	○ ألكثير	○ ألقليل	○ لست متأكد(ة)
العنب	○ ألكثير	○ ألقليل	○ لست متأكد(ة)

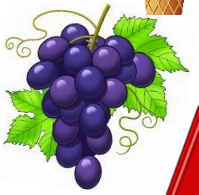

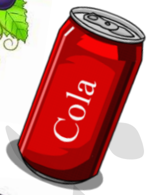

8. **برأيك، هل تحتوي المأكولات التالية على الكثير او القليل من الدسم؟**

معكرونة (دون صلصة)	○ ألكثير	○ ألقليل	○ لست متأكد(ة)
البقوليات (عدس،فاصوليا...)	○ ألكثير	○ ألقليل	○ لست متأكد(ة)
عسل	○ ألكثير	○ ألقليل	○ لست متأكد(ة)
البذورات	○ ألكثير	○ ألقليل	○ لست متأكد(ة)
خبز	○ ألكثير	○ ألقليل	○ لست متأكد(ة)
جبنة الشيدار	○ ألكثير	○ ألقليل	○ لست متأكد(ة)
شيبس	○ ألكثير	○ ألقليل	○ لست متأكد(ة)
جبنة القشقوان	○ ألكثير	○ ألقليل	○ لست متأكد(ة)
مرتديلا	○ ألكثير	○ ألقليل	○ لست متأكد(ة)

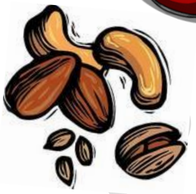

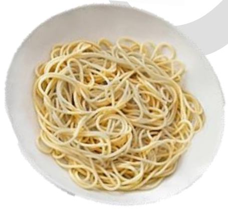

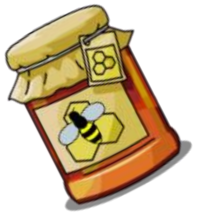

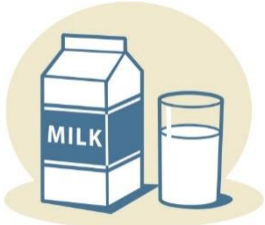

9. برأيك، هل تحتوي المأكولات التالية على الكثير او القليل من الدهون المشبعة؟

حليب كامل الدسم	○ ألكثير	○ ألقليل	○ لست متأكد(ة)
زيت زيتون	○ ألكثير	○ ألقليل	○ لست متأكد(ة)
السمنة	○ ألكثير	○ ألقليل	○ لست متأكد(ة)
شوكولا (كريما)	○ ألكثير	○ ألقليل	○ لست متأكد(ة)

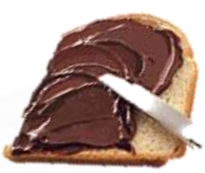

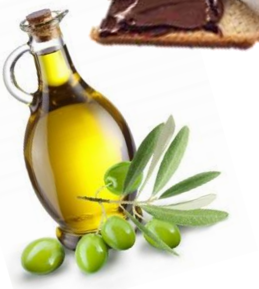

10. برأيك، هل تحتوي المأكولات التالية على الكثير او القليل من الملح؟

هوت دوغ	○ ألكثير	○ ألقليل	○ لست متأكد(ة)
اللحوم	○ ألكثير	○ ألقليل	○ لست متأكد(ة)
خضار طازجة	○ ألكثير	○ ألقليل	○ لست متأكد(ة)
شيبس	○ ألكثير	○ ألقليل	○ لست متأكد(ة)
بطاطا مقلية (جاهزة)	○ ألكثير	○ ألقليل	○ لست متأكد(ة)

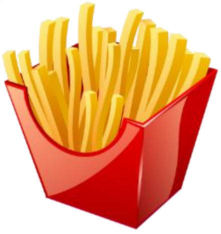

11. برأيك، هل تحتوي المأكولات التالية على الكثير او القليل من البروتين؟

دجاج	○ ألكثير	○ ألقليل	○ لست متأكد(ة)
جبنة	○ ألكثير	○ ألقليل	○ لست متأكد(ة)
فاكهة	○ ألكثير	○ ألقليل	○ لست متأكد(ة)
بقوليات (عدس، فاصوليا...)	○ ألكثير	○ ألقليل	○ لست متأكد(ة)
زبدة	○ ألكثير	○ ألقليل	○ لست متأكد(ة)
بيض	○ ألكثير	○ ألقليل	○ لست متأكد(ة)

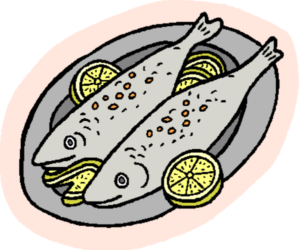

12. برأيك، هل تحتوي المأكولات التالية على الكثير او القليل من الألياف؟

بيض	○ ألكثير	○ ألقليل	○ لست متأكد(ة)
لحم البقر	○ ألكثير	○ ألقليل	○ لست متأكد(ة)
بروكولي	○ ألكثير	○ ألقليل	○ لست متأكد(ة)
بذورات	○ ألكثير	○ ألقليل	○ لست متأكد(ة)
سمك	○ ألكثير	○ ألقليل	○ لست متأكد(ة)
بقوليات (عدس، فاصوليا...)	○ ألكثير	○ ألقليل	○ لست متأكد(ة)
ارز	○ ألكثير	○ ألقليل	○ لست متأكد(ة)
برغل	○ ألكثير	○ ألقليل	○ لست متأكد(ة)

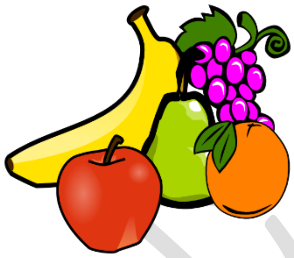

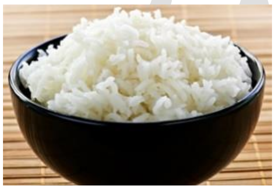

13. بعض المأكولات الغنية بالدهون لا تحتوي على الكولسترول:

○ صح ○ خطأ ○ لست متأكد(ة)

14. السكر الاسمر هو البديل الصحي للسكر الابيض:

○ صح ○ خطأ ○ لست متأكد(ة)

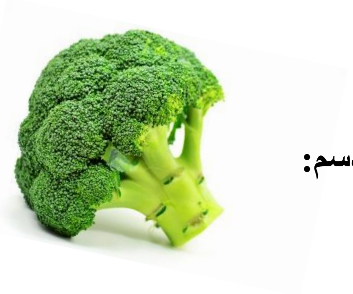

15. يحتوي كوب الحليب الكامل الدسم على بروتينات أكثر من الحليب الخالي من الدسم:

○ صح ○ خطأ ○ لست متأكد(ة)

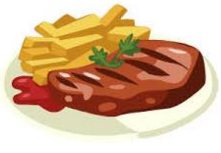 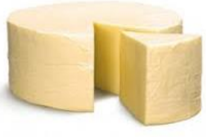 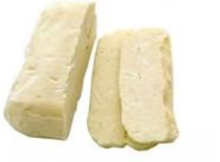 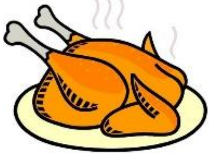 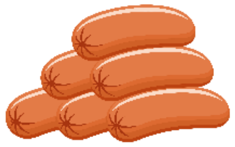

16. أي من الزيوت التالية تحتوي على الكمية الأكبر من الزيوت الغير مشبعة (إختر إجابة واحدة)

- ○ زيت الزيتون ○ زبدة ○ زيت النخيل ○ لست متأكد(ة)

17. ما هو الخبز الذي يحتوي علئ الكمية الأكبر من الفيتامينات والمعادن (إختر إجابة واحدة)

- ○ الخبز الابيض ○ الخبز الاسمر ○ لست متأكد(ة)

18. يحتوي كوب الحليب الكامل الدسم علئ كالسيوم اكثر مقارنة بالحليب الخالي من الدسم (إختر إجابة واحدة)

- ○ صح ○ خطأ ○ لست متأكد(ة)

19. لو أراد احدٌ تخفيض كمية الدسم في طعامه، ما هو الخيار الأنسب؟

- ○ستيك مشوي ○ سفينة دجاج مشوية ○سجق ○ لحم غنم مشوي ○ لست متأكد(ة)

20. ما هي الجبنة التي تحتوي على الكمية الأقل من الدسم؟

- ○ قشقوان ○ عكاوي ○حلوم ○ جبنة الكريما (بيكون، كيري...) ○ لست متأكد(ة)

21. برأيك، ما الذي سيساعد على الوقاية من أمراض القلب؟

تناول الياف اكثر	○ نعم	○ لا	○ لست متأكد(ة)
تناول ملح أقل	○ نعم	○ لا	○ لست متأكد(ة)
تناول دهون مشبعة أكثر	○ نعم	○ لا	○ لست متأكد(ة)

22. خطر الإصابة بالعديد من الأمراض المزمنة (كالسكري وأمراض القلب وبعض أمراض السرطان) متعلق بتناول القليل من الخضار والفاكهة:

- ○ صح ○ خطأ ○ لست متأكد(ة)

23. ألتدخين يسبب العديد من الأمراض المزمنة (كسرطان الرئة وامراض القلب) :

- ○ صح ○ خطأ ○ لست متأكد(ة)

شكراً جزيلاً لملئ الإستبيان.

ان كان لديك اية اراء حول الإستبيان، الرجاء كتابتها على الأسطر أدناه.

____

____

Adopted from: Parmenter and Wardle, European Journal of Clinical Nutrition, 53, 298-308, 1999.

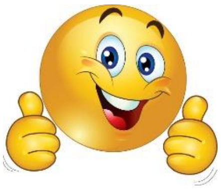

صحتك بصحتك
