# Supplementary material for: Development of Dietary Knowledge and Adherence Questionnaires for Lebanese Adolescents and Their Parents
Source: Int J Environ Res Public Health. 2019 Dec 24;17(1):147. doi: 10.3390/ijerph17010147 (PMC6982025; doi:10.3390/ijerph17010147)

# Dietary Knowledge Questionnaire

حضرة الأهالي الأعزاء،

- ألرجاء تعبئة الإستبيان التالي بشأن العادات الغذائية والرياضة كجزء من بحث علمي تجريه جامعة ماسترشت في هولندا خلال العام الدراسي 2017-2016.
- تهدف هذه الدراسة إلى تقييم السلوك الغذائي لأهالي المراهقين اللبنانيين وأولادهم.
- سيطلب منكم الإجابة على 23 سؤال حول المعرفة الغذائية، وذلك لمدة 15 دقيقة.
- يمكنك التوقف عن المشاركة في هذا البحث خلال اي وقت. إن المشاركة في هذا المشروع هي طوعية.
- ستكون جميع الإستبيانات مجهولة الهوية، وبالتالي لن يتم الأفصاح عن اية اجوبة.
- للمزيد من المعلومات، لا تتردد بطرح الأسئلة.
- من خلال تعبئة هذا الإستبيان، إنك توافق على المشاركة في هذا المشروع.

شكرً لدعمكم.

– **الرقم:**________________________
– **العمر:** ________
– **شهر الولادة:**

| | | | |
|---|---|---|---|
| ○ كانون الثاني | ○ شباط | ○ اذار | ○ نيسان |
| ○ ايار | ○ حزيران | ○ تموز | ○ اب |
| ○ ايلول | ○ تشرين الاول | ○ تشرين الثاني | ○ كانون الاول |

– **سنة الولادة:**
– **رجل/امرأة:**
○ رجل ○ امرأة

– **العنوان (المدينة/القرية):** ___________

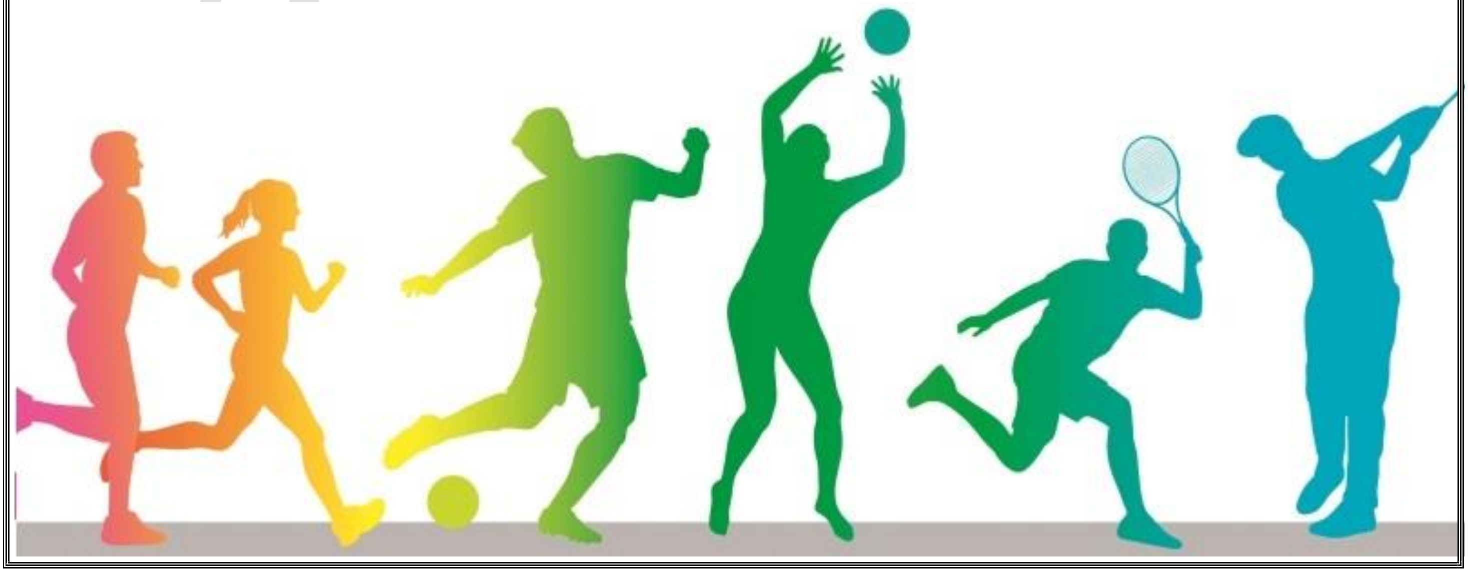

1. كم حصة من الخضار والفاكهة ينصح بتناولها خلال النهار؟
   - ○ 0-1 ○ 2-4 ○ 5 أو أكثر ○ لست متأكد(ة)

2. ما نوع الدسم الذي يجب تجنبه؟
   - ○ الدهون الغير مشبعة ○ الدهون المشبعة ○ لست متأكد(ة)

3. أي من مشتقات الحليب التالية يجب تناولها أكثر من غيرها؟
   - ○ مشتقات الحليب الكاملة الدسم ○ مشتقات الحليب القليلة الدسم ○ علينا تناول مشتقات الحليب الكاملة الدسم والقليلة الدسم ○ علينا تجنب تناول مشتقات الحليب ○ لست متأكد(ة)

4. كم كوب من الماء ينصح بالشرب خلال النهار؟
   - ○ 1-3 ○ 4-7 ○ 8 ○ لست متأكد(ة)

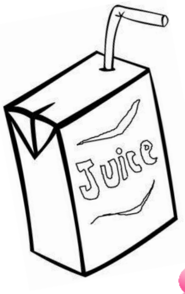


5. لكم من الوقت ينصح بممارسة الرياضة يومياً؟
   - ○ 10-15دقائق ○ 15-20 دقيقة ○ 30 دقيقة أو أكثر ○ لست متأكد(ة)

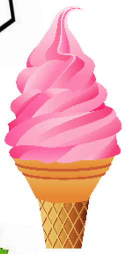

6. كم كوب من مشروب الطاقة يسمح بالشرب خلال النهار؟
   - ○ 0 ○ 1-2 ○ 3-4 ○ لست متأكد(ة)

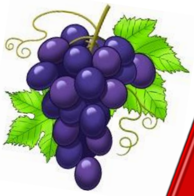

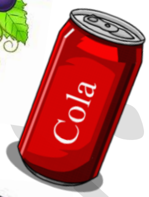


7. برأيك، هل تحتوي المأكولات التالية على الكثير أو القليل من السكر المضاف؟

| | | | |
|---|---|---|---|
| التفاح | ○ ألكثير | ○ ألقليل | ○ لست متأكد(ة) |
| المثلجات | ○ ألكثير | ○ ألقليل | ○ لست متأكد(ة) |
| عصير الفاكهة المصنع | ○ ألكثير | ○ ألقليل | ○ لست متأكد(ة) |
| المشروبات الغازية | ○ ألكثير | ○ ألقليل | ○ لست متأكد(ة) |
| العنب | ○ ألكثير | ○ ألقليل | ○ لست متأكد(ة) |

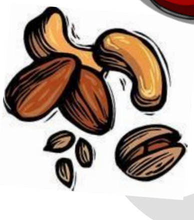

8. برأيك، هل تحتوي المأكولات التالية على الكثير او القليل من الدسم؟

| | | | |
|---|---|---|---|
| معكرونة (دون صلصة) | ○ ألكثير | ○ ألقليل | ○ لست متأكد(ة) |
| البقوليات (عدس،فاصوليا...) | ○ ألكثير | ○ ألقليل | ○ لست متأكد(ة) |
| عسل | ○ ألكثير | ○ ألقليل | ○ لست متأكد(ة) |
| البذورات | ○ ألكثير | ○ ألقليل | ○ لست متأكد(ة) |
| خبز | ○ ألكثير | ○ ألقليل | ○ لست متأكد(ة) |
| جبنة الشيدار | ○ ألكثير | ○ ألقليل | ○ لست متأكد(ة) |
| شيبس | ○ ألكثير | ○ ألقليل | ○ لست متأكد(ة) |
| جبنة القشقوان | ○ ألكثير | ○ ألقليل | ○ لست متأكد(ة) |
| مرتديلا | ○ ألكثير | ○ ألقليل | ○ لست متأكد(ة) |

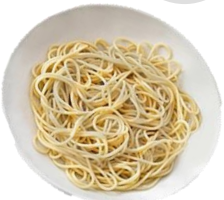

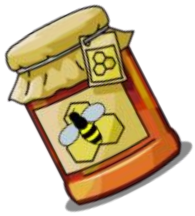

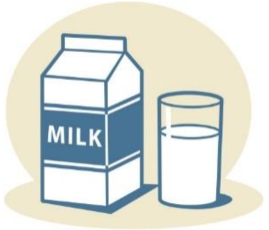


**9. برأيك، هل تحتوي المأكولات التالية على الكثير او القليل من الدهون المشبعة؟**

| | | | |
|---|---|---|---|
| حليب كامل الدسم | ○ ألكثير | ○ ألقليل | ○ لست متأكد(ة) |
| زيت زيتون | ○ ألكثير | ○ ألقليل | ○ لست متأكد(ة) |
| السمنة | ○ ألكثير | ○ ألقليل | ○ لست متأكد(ة) |
| شوكولا (كريما) | ○ ألكثير | ○ ألقليل | ○ لست متأكد(ة) |

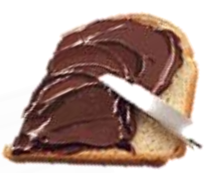

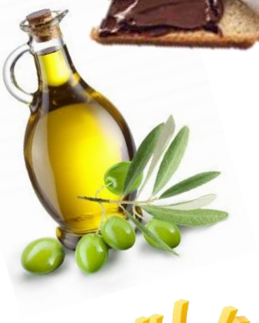

**10. برأيك، هل تحتوي المأكولات التالية على الكثير او القليل من الملح؟**

| | | | |
|---|---|---|---|
| هوت دوغ | ○ ألكثير | ○ ألقليل | ○ لست متأكد(ة) |
| اللحوم | ○ ألكثير | ○ ألقليل | ○ لست متأكد(ة) |
| خضار طازجة | ○ ألكثير | ○ ألقليل | ○ لست متأكد(ة) |
| شيبس | ○ ألكثير | ○ ألقليل | ○ لست متأكد(ة) |
| بطاطا مقلية (جاهزة) | ○ ألكثير | ○ ألقليل | ○ لست متأكد(ة) |

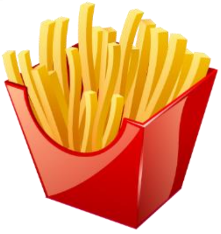

**11. برأيك، هل تحتوي المأكولات التالية على الكثير او القليل من البروتين؟**

| | | | |
|---|---|---|---|
| دجاج | ○ ألكثير | ○ ألقليل | ○ لست متأكد(ة) |
| جبنة | ○ ألكثير | ○ ألقليل | ○ لست متأكد(ة) |
| فاكهة | ○ ألكثير | ○ ألقليل | ○ لست متأكد(ة) |
| بقوليات (عدس، فاصوليا...) | ○ ألكثير | ○ ألقليل | ○ لست متأكد(ة) |
| زبدة | ○ ألكثير | ○ ألقليل | ○ لست متأكد(ة) |
| بيض | ○ ألكثير | ○ ألقليل | ○ لست متأكد(ة) |

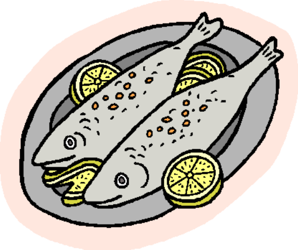

**12. برأيك، هل تحتوي المأكولات التالية على الكثير او القليل من الألياف؟**

| | | | |
|---|---|---|---|
| بيض | ○ ألكثير | ○ ألقليل | ○ لست متأكد(ة) |
| لحم البقر | ○ ألكثير | ○ ألقليل | ○ لست متأكد(ة) |
| بروكولي | ○ ألكثير | ○ ألقليل | ○ لست متأكد(ة) |
| بذورات | ○ ألكثير | ○ ألقليل | ○ لست متأكد(ة) |
| سمك | ○ ألكثير | ○ ألقليل | ○ لست متأكد(ة) |
| بقوليات (عدس، فاصوليا...) | ○ ألكثير | ○ ألقليل | ○ لست متأكد(ة) |
| ارز | ○ ألكثير | ○ ألقليل | ○ لست متأكد(ة) |
| برغل | ○ ألكثير | ○ ألقليل | ○ لست متأكد(ة) |

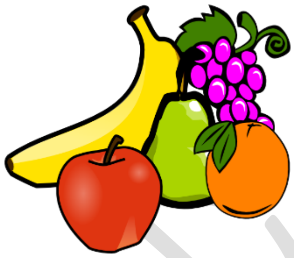

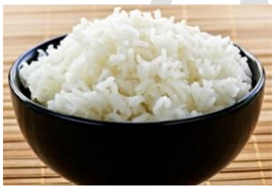

**13. بعض المأكولات الغنية بالدهون لا تحتوي على الكولسترول:**

○ صح ○ خطأ ○ لست متأكد(ة)

**14. السكر الاسمر هو البديل الصحي للسكر الابيض:**

○ صح ○ خطأ ○ لست متأكد(ة)

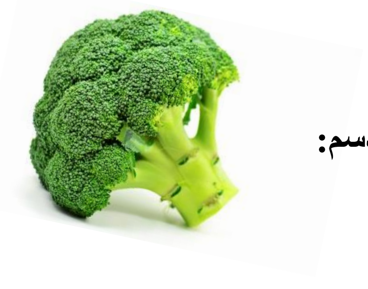

**15. يحتوي كوب الحليب الكامل الدسم على بروتينات أكثر من الحليب الخالي من الدسم:**

○ صح ○ خطأ ○ لست متأكد(ة)

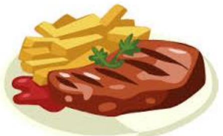 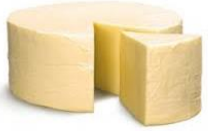 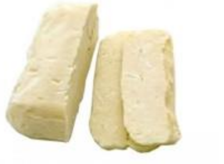 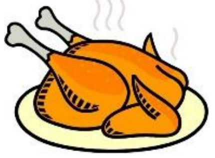 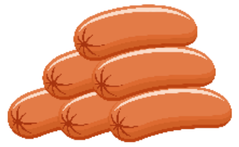

**16. أي من الزيوت التالية تحتوي على الكمية الأكبر من الزيوت الغير مشبعة (إختر إجابة واحدة)**
- o زيت الزيتون  o زبدة  o زيت النخيل  o لست متأكد(ة)

**17. ما هو الخبز الذي يحتوي علئ الكمية الأكبر من الفيتامينات والمعادن (إختر إجابة واحدة)**
- o الخبز الابيض  o الخبز الاسمر  o لست متأكد(ة)

**18. يحتوي كوب الحليب الكامل الدسم علئ كالسيوم اكثر مقارنة بالحليب الخالي من الدسم (إختر إجابة واحدة)**
- o صح  o خطأ  o لست متأكد(ة)

**19. لو أراد احدٌ تخفيض كمية الدسم في طعامه، ما هو الخيار الأنسب؟**
- o ستيك مشوي  o سفينة دجاج مشوية  o سجق  o لحم غنم مشوي  o لست متأكد(ة)

**20. ما هي الجبنة التي تحتوي على الكمية الأقل من الدسم؟**
- o قشقوان  o عكاوي  o حلوم  o جبنة الكريما (بيكون، كيري...)  o لست متأكد(ة)

**21. برأيك، ما الذي سيساعد على الوقاية من أمراض القلب؟**

| | | | |
|---|---|---|---|
| تناول الياف اكثر | o نعم | o لا | o لست متأكد(ة) |
| تناول ملح أقل | o نعم | o لا | o لست متأكد(ة) |
| تناول دهون مشبعة أكثر | o نعم | o لا | o لست متأكد(ة) |

**22. خطر الإصابة بالعديد من الأمراض المزمنة (كالسكري وأمراض القلب وبعض أمراض السرطان) متعلق بتناول القليل من الخضار والفاكهة:**
- o صح  o خطأ  o لست متأكد(ة)

**23. ألتدخين يسبب العديد من الأمراض المزمنة (كسرطان الرئة وامراض القلب):**
- o صح  o خطأ  o لست متأكد(ة)

**شكراً جزيلاً لملئ الإستبيان.**

ان كان لديك اية اراء حول الإستبيان، الرجاء كتابتها على الأسطر أدناه.

Adopted from: Parmenter and Wardle, European Journal of Clinical Nutrition, 53, 298-308, 1999.

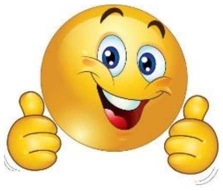

Supplement: Supplementary file 1 [file ijerph-17-00147-s001.zip › Supplementary material - S4 - DKQ parents Ar.pdf]
