# Supplementary material for: Development of Dietary Knowledge and Adherence Questionnaires for Lebanese Adolescents and Their Parents
Source: Int J Environ Res Public Health. 2019 Dec 24;17(1):147. doi: 10.3390/ijerph17010147 (PMC6982025; doi:10.3390/ijerph17010147)
Supplement: Supplementary file 1 [file ijerph-17-00147-s001.zip › Supplementary material - S5- DAQ En.pdf]

Name:

Dietary Adherence Questionnaire

- **ID:**
- **School' name:**
- **Age:**
- **Birth month:**
  - January
  - February
  - March
  - April
  - May
  - June
  - July
  - August
  - September
  - October
  - November
  - December
- **Birth year:**
  - 1999
  - 2000
  - 2001
  - Other: ______
- **Grade:**
  - Grade 10
  - Grade 11
  - Grade 12
- **Boy/girl:**
  - Boy
  - Girl
- **Address (city/ village):**

- You are invited to participate in a scientific research conducted by Maastricht University in the Netherlands for the academic year 2016-2017.
- The purpose of this study is to assess the dietary behavior of Lebanese adolescents
- You will be asked to answer 30 questions regarding dietary adherence to nutritional recommendations. Completing the questionnaire will take approximately 20 minutes.
- You are free to withdraw at any time. Participation is voluntary.
- All the collected data will be anonymous, and answers will not be revealed.
- Feel free to ask any additional question.
- By filling this questionnaire, you agree to participate in this project.

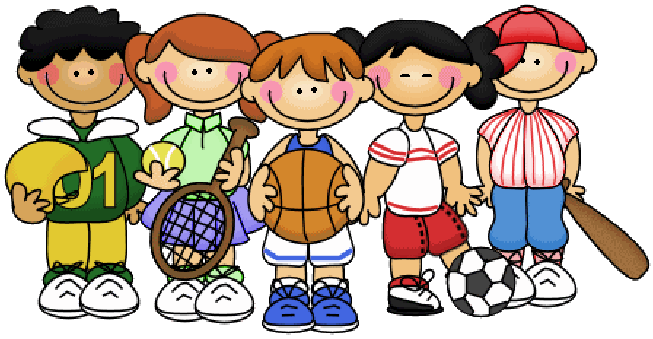

Sahtak bi Sahnak

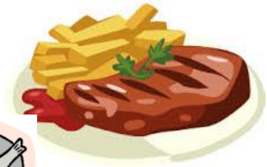
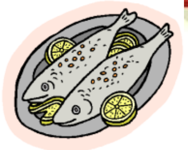
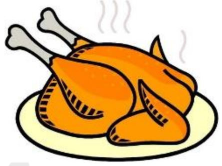

1. **Yesterday, did you eat meat (like chopped meat in stews), chicken breast (grilled/boiled/not fried), or fish (grilled/boiled/not fried)?**
    a. No, I did not eat any of the foods listed above
    b. Yes, I ate one of these foods once
    c. Yes, I ate one of these foods twice
    d. Yes, I ate one of these foods 3 times or more

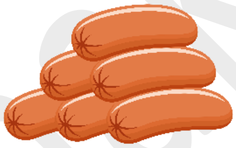

2. **Yesterday, did you eat fried chicken, chicken nuggets, fried fish, fried meat, hot dogs, sausage, mortadella, or ham?**
    a. No, I did not eat any of the foods listed above
    b. Yes, I ate one of these foods once
    c. Yes, I ate one of these foods twice
    d. Yes, I ate one of these foods 3 times or more

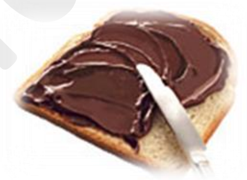
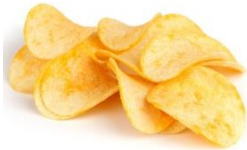

3. **Yesterday, did you eat chocolate cream or potato chips?**
    a. No, I did not eat any of the foods listed above
    b. Yes, I ate one of these foods once
    c. Yes, I ate one of these foods twice
    d. Yes, I ate one of these foods 3 times or more

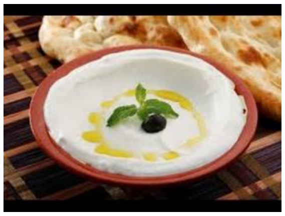

4. **Yesterday, did you eat any of these foods? Labneh, shanklish, kareesha**
    a. No, I did not eat any of the foods listed above
    b. Yes, I ate one of these foods once
    c. Yes, I ate one of these foods twice
    d. Yes, I ate one of these foods 3 times or more

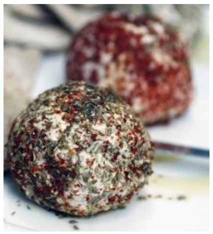

5. **Yesterday, did you eat any of these cheeses? Mozzarella, feta, akawi, baladiye, khadra**
    a. No, I did not eat any of the foods listed above
    b. Yes, I ate one of these foods once
    c. Yes, I ate one of these foods twice
    d. Yes, I ate one of these foods 3 times or more

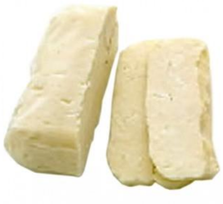

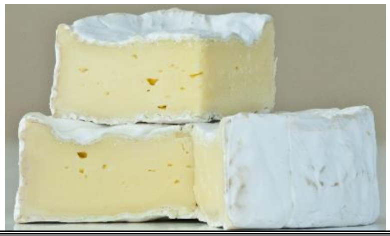
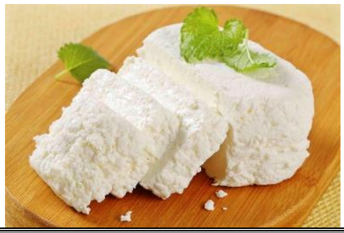
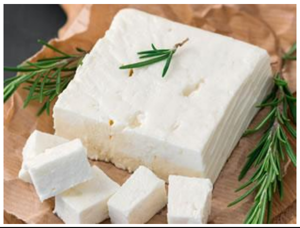
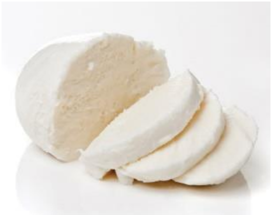

Sahtak bi Sahnak

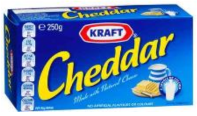

6. **Yesterday, did you eat any of these foods? Cheddar cheese, gruyere, edam, goat cheese, gouda, parmesan, Roquefort, kashkawan, cream cheese, kishk**
    a. No, I did not eat any of the foods listed above
    b. Yes, I ate one of these foods once
    c. Yes, I ate one of these foods twice
    d. Yes, I ate one of these foods 3 times or more

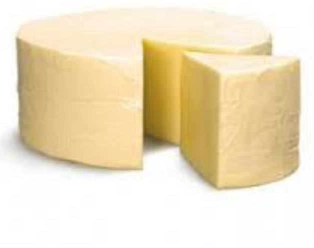

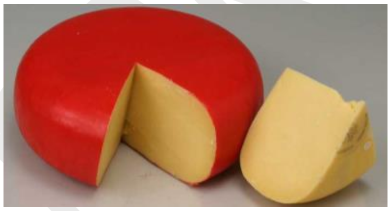

7. **Yesterday, did you drink milk or laban?**
    a. No, I did not eat any of the foods listed above
    b. Yes, I ate one of these foods once
    c. Yes, I ate one of these foods twice
    d. Yes, I ate one of these foods 3 times or more

8. **Yesterday, did you drink skimmed milk (reduced or 0% fat) or skimmed laban?**
    a. No, I did not eat any of the foods listed above
    b. Yes, I ate one of these foods once
    c. Yes, I ate one of these foods twice
    d. Yes, I ate one of these foods 3 times or more

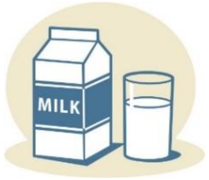

9. **Yesterday, did you eat white Arabic bread, kaak, franjeh bread?**
    a. No, I did not eat any of the foods listed above
    b. Yes, I ate one of these foods once
    c. Yes, I ate one of these foods twice
    d. Yes, I ate one of these foods 3 times or more

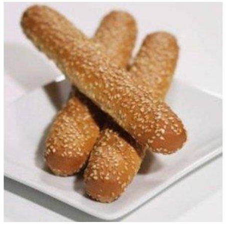

10. **Yesterday, did you eat whole bread, oats bread, brown bread, tortillas?**
    a. No, I did not eat any of the foods listed above
    b. Yes, I ate one of these foods once
    c. Yes, I ate one of these foods twice
    d. Yes, I ate one of these foods 3 times or more

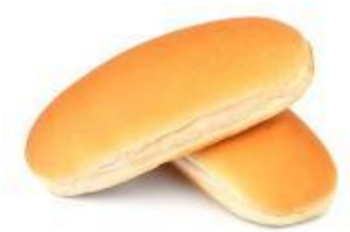

11. **Yesterday, did you eat French fries (fried potatoes)?**
    a. No, I did not eat any of the foods listed above
    b. Yes, I ate one of these foods once
    c. Yes, I ate one of these foods twice
    d. Yes, I ate one of these foods 3 times or more

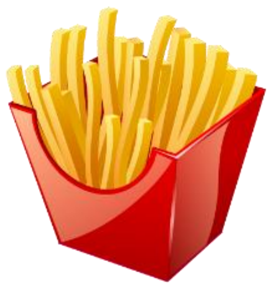

12. **Yesterday, did you eat beans like lentils, white beans, fava beans (do not count green beans)?**
    a. No, I did not eat any of the foods listed above
    b. Yes, I ate one of these foods once
    c. Yes, I ate one of these foods twice
    d. Yes, I ate one of these foods 3 times or more

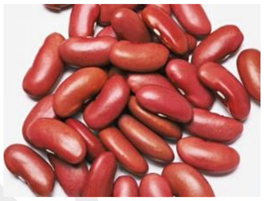

13. **Yesterday, did you eat any vegetables (do not count potatoes)?**
    a. No, I did not eat any of the foods listed above
    b. Yes, I ate one of these foods once
    c. Yes, I ate one of these foods twice
    d. Yes, I ate one of these foods 3 times or more

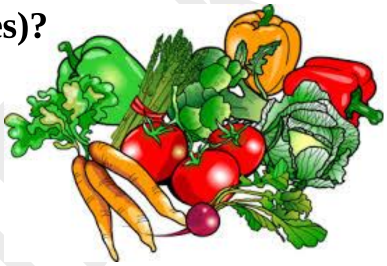

14. **Yesterday, did you eat fresh fruits? Do not count fruit juice and dried fruits.**
    a. No, I did not eat any of the foods listed above
    b. Yes, I ate one of these foods once
    c. Yes, I ate one of these foods twice
    d. Yes, I ate one of these foods 3 times or more

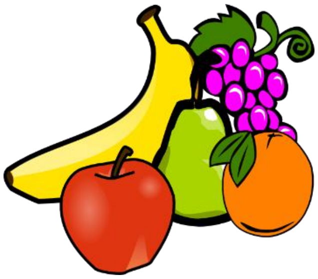

15. **Yesterday, did you drink natural fruit juice?**
    a. No, I did not eat any of the foods listed above
    b. Yes, I ate one of these foods once
    c. Yes, I ate one of these foods twice
    d. Yes, I ate one of these foods 3 times or more

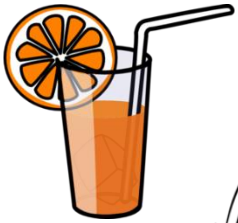

16. **Yesterday, did you drink commercial fruit juice?**
    a. No, I did not eat any of the foods listed above
    b. Yes, I ate one of these foods once
    c. Yes, I ate one of these foods twice
    d. Yes, I ate one of these foods 3 times or more

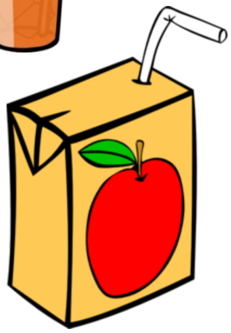

17. **Yesterday, did you drink soft drinks (Pepsi, 7up, Miranda, Sprite, Coca Cola, Fanta…)?**
    a. No, I did not eat any of the foods listed above
    b. Yes, I ate one of these foods once
    c. Yes, I ate one of these foods twice
    d. Yes, I ate one of these foods 3 times or more

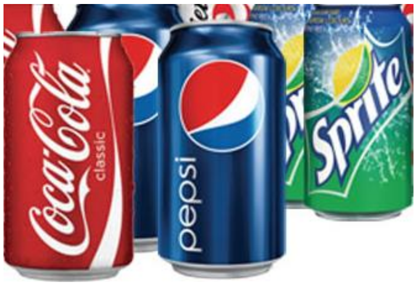

18. Yesterday, did you drink <u>diet</u> soft drinks (Pepsi diet, 7up diet, Coca Cola diet, …)?

a. No, I did not eat any of the foods listed above
b. Yes, I ate one of these foods once
c. Yes, I ate one of these foods twice
d. Yes, I ate one of these foods 3 times or more

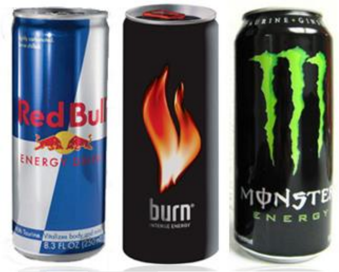

19. Yesterday, did you drink energy drinks (Red Bull, AMP, …)?

a. No, I did not eat any of the foods listed above
b. Yes, I ate one of these foods once
c. Yes, I ate one of these foods twice
d. Yes, I ate one of these foods 3 times or more

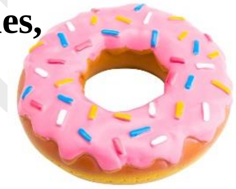

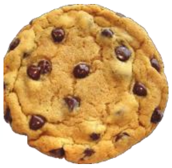

20. Yesterday, did you eat any sweets such as sweet rolls, cookies, cakes, pies, brownies, cheesecake, doughnuts?

a. No, I did not eat any of the foods listed above
b. Yes, I ate one of these foods once
c. Yes, I ate one of these foods twice
d. Yes, I ate one of these foods 3 times or more

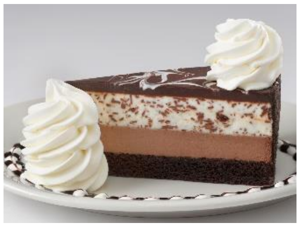

21. Yesterday, did you eat any Arabic sweets (namoura, knefeh, halewet el jibn, znood el sitt…)?

a. No, I did not eat any of the foods listed above
b. Yes, I ate one of these foods once
c. Yes, I ate one of these foods twice
d. Yes, I ate one of these foods 3 times or more

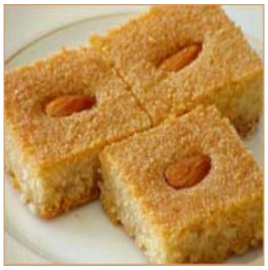

22. Yesterday, did you have breakfast?

a. Yes   b. No

23. Yesterday, how many meals did you eat (meals include breakfast, lunch, and dinner)?

a. I did not have any meal
b. I had 1 meal
c. I had 2 meals
d. I had 3 or more meals

24. Yesterday, did you have a snack? A snack is a food or drink (except for water) that you eat or drink between meals.

a. No, I did not eat any snack
b. Yes, I ate a snack once
c. Yes, I ate a snack twice
d. Yes, I ate a snack 3 times or more

25. Yesterday, how many times did you eat food from outside of your house? (restaurants, fast food restaurants, pizza places and cafeterias)

a. No, I did not eat outside of the house
b. Yes, I had 1 meal
c. Yes, I had 2 meals
d. Yes, I had 3 or more meals

26. Do you participate in physical education sessions at school?

a. Yes b. No

27. Do you participate in any sports activity (other than physical education session at schools)?

a. Yes b. No

28. How many hours per week do you exercise?

a. 0 b. 1-2 c. 3-4 d. >5

29. How many hours per day do you usually spend playing video games, watching TV, using tablets and chatting on the phone?

a. 0hour b. 1hour c. 2hours d. 3hours e. 4hours f. 5hours g. 6hours h. >6hours

30. Do you smoke? (cigarettes and narjileh are included)

a. Yes b. No c. Used to smoke

Adapted from SPANS 2010

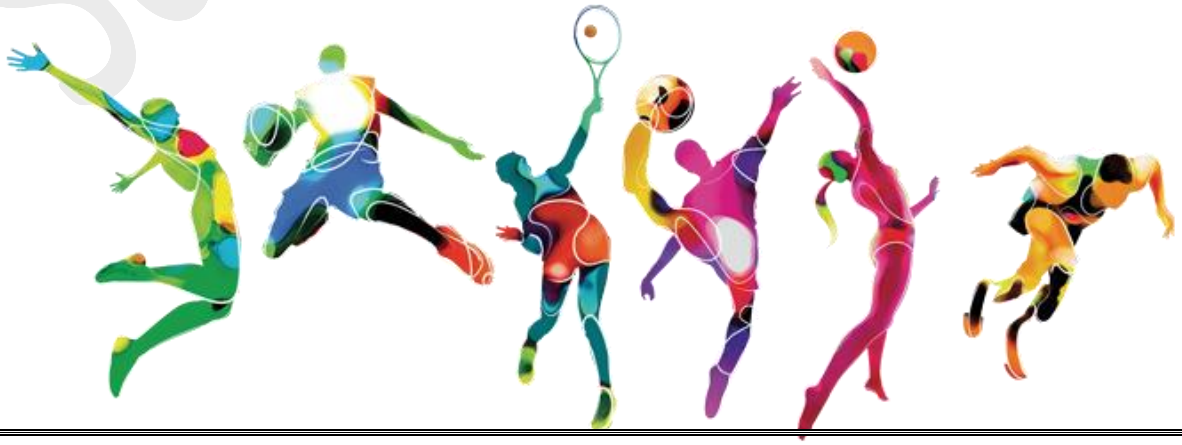

Sahtak bi Sahnak
