# Supplementary material for: Development of Dietary Knowledge and Adherence Questionnaires for Lebanese Adolescents and Their Parents
Source: Int J Environ Res Public Health. 2019 Dec 24;17(1):147. doi: 10.3390/ijerph17010147 (PMC6982025; doi:10.3390/ijerph17010147)
Supplement: Supplementary file 1 [file ijerph-17-00147-s001.zip › Supplementary material - S7 - DAQ parents En.pdf]

Dietary Adherence Questionnaire

Dear parents,

- You are invited to participate in a scientific research conducted by Maastricht University in the Netherlands for the academic year 2016-2017.
- The purpose of this study is to assess the dietary behavior of Lebanese adolescents.
- You will be asked to answer 29 questions regarding dietary adherence to nutritional recommendations. Completing the questionnaire will take approximately 20 minutes.
- You are free to withdraw at any time. Participation is voluntary.
- All the collected data will be anonymous, and answers will not be revealed.
- Feel free to ask any additional question.
- By filling this questionnaire, you agree to participate in this project.

Thank you for your support.

- **ID:**
- **Age:**
- **Birth month:**
  - ○ January ○ April ○ July ○ October
  - ○ February ○ May ○ August ○ November
  - ○ March ○ June ○ September ○ December
- **Birth year:**
- **Man/woman:**
  - ○ Man ○ Woman
- **Address (city/ village):**

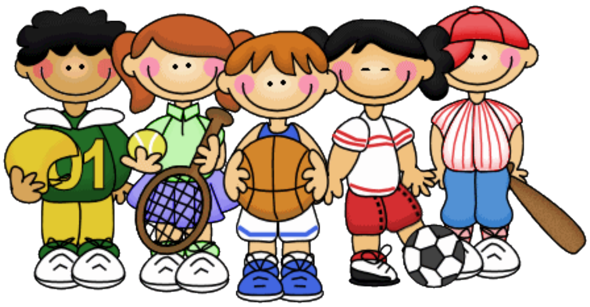

Sahtak bi Sahnak

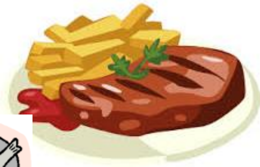
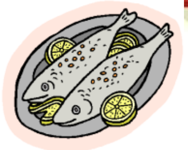
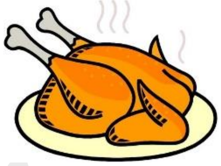

1. **Yesterday, did you eat meat (like chopped meat in stews), chicken breast (grilled/boiled/not fried), or fish (grilled/boiled/not fried)?**
    a. No, I did not eat any of the foods listed above
    b. Yes, I ate one of these foods once
    c. Yes, I ate one of these foods twice
    d. Yes, I ate one of these foods 3 times or more

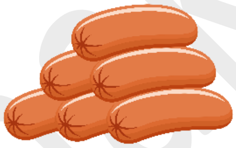

2. **Yesterday, did you eat fried chicken, chicken nuggets, fried fish, fried meat, hot dogs, sausage, mortadella, or ham?**
    a. No, I did not eat any of the foods listed above
    b. Yes, I ate one of these foods once
    c. Yes, I ate one of these foods twice
    d. Yes, I ate one of these foods 3 times or more

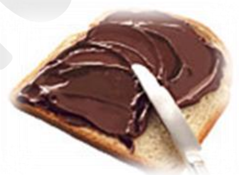
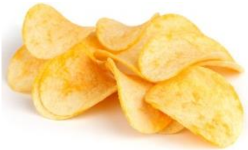

3. **Yesterday, did you eat chocolate cream or potato chips?**
    a. No, I did not eat any of the foods listed above
    b. Yes, I ate one of these foods once
    c. Yes, I ate one of these foods twice
    d. Yes, I ate one of these foods 3 times or more

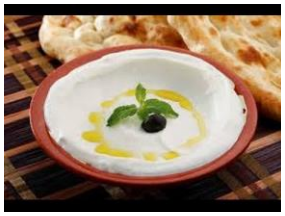

4. **Yesterday, did you eat any of these foods? Labneh, shanklish, kareesha**
    a. No, I did not eat any of the foods listed above
    b. Yes, I ate one of these foods once
    c. Yes, I ate one of these foods twice
    d. Yes, I ate one of these foods 3 times or more

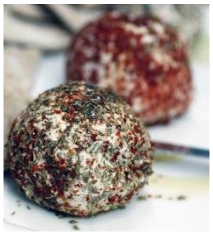

5. **Yesterday, did you eat any of these cheeses? Mozzarella, feta, akawi, baladiye, khadra**
    a. No, I did not eat any of the foods listed above
    b. Yes, I ate one of these foods once
    c. Yes, I ate one of these foods twice
    d. Yes, I ate one of these foods 3 times or more

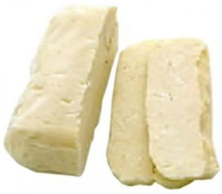

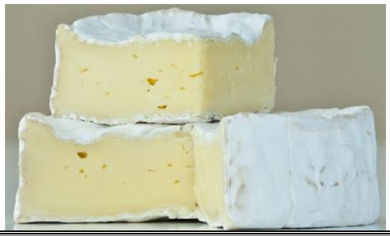
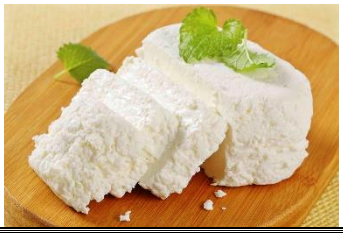
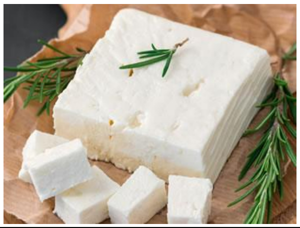
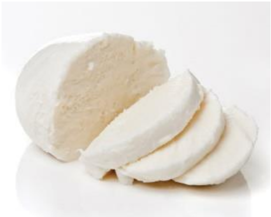

Sahtak bi Sahnak

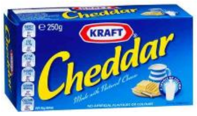

6. **Yesterday, did you eat any of these foods? Cheddar cheese, gruyere, edam, goat cheese, gouda, parmesan, Roquefort, kashkawan, cream cheese, kishk**
    a. No, I did not eat any of the foods listed above
    b. Yes, I ate one of these foods once
    c. Yes, I ate one of these foods twice
    d. Yes, I ate one of these foods 3 times or more

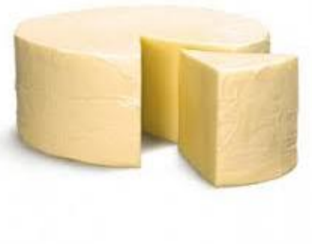

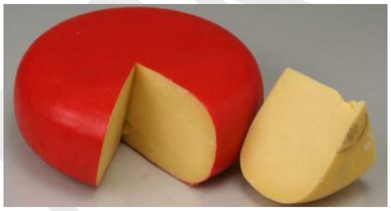

7. **Yesterday, did you drink milk or laban?**
    a. No, I did not eat any of the foods listed above
    b. Yes, I ate one of these foods once
    c. Yes, I ate one of these foods twice
    d. Yes, I ate one of these foods 3 times or more

8. **Yesterday, did you drink skimmed milk (reduced or 0% fat) or skimmed laban?**
    a. No, I did not eat any of the foods listed above
    b. Yes, I ate one of these foods once
    c. Yes, I ate one of these foods twice
    d. Yes, I ate one of these foods 3 times or more

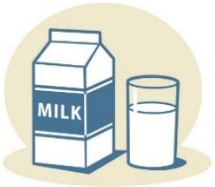

9. **Yesterday, did you eat white arabic bread, kaak, franjeh bread?**
    a. No, I did not eat any of the foods listed above
    b. Yes, I ate one of these foods once
    c. Yes, I ate one of these foods twice
    d. Yes, I ate one of these foods 3 times or more

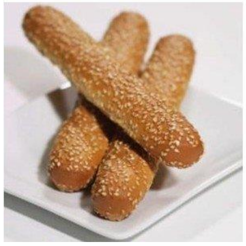

10. **Yesterday, did you eat whole bread, oats bread, brown bread, tortillas?**
    a. No, I did not eat any of the foods listed above
    b. Yes, I ate one of these foods once
    c. Yes, I ate one of these foods twice
    d. Yes, I ate one of these foods 3 times or more

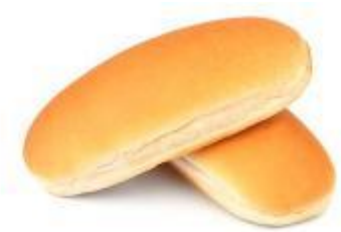

11. **Yesterday, did you eat French fries (fried potatoes)?**
    a. No, I did not eat any of the foods listed above
    b. Yes, I ate one of these foods once
    c. Yes, I ate one of these foods twice
    d. Yes, I ate one of these foods 3 times or more

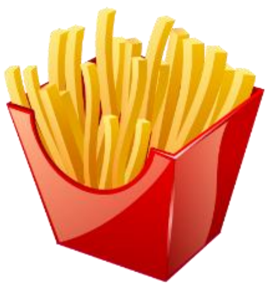

12. Yesterday, did you eat beans like lentils, white beans, fava beans (do not count green beans)?

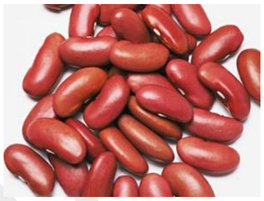

a. No, I did not eat any of the foods listed above
b. Yes, I ate one of these foods once
c. Yes, I ate one of these foods twice
d. Yes, I ate one of these foods 3 times or more

13. Yesterday, did you eat any vegetables (do not count potatoes)?

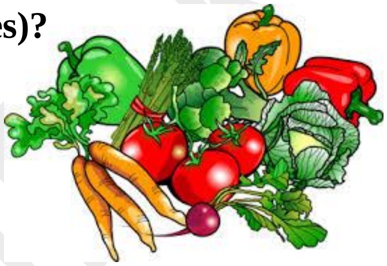

a. No, I did not eat any of the foods listed above
b. Yes, I ate one of these foods once
c. Yes, I ate one of these foods twice
d. Yes, I ate one of these foods 3 times or more

14. Yesterday, did you eat fresh fruits? Do not count fruit juice and dried fruits.

a. No, I did not eat any of the foods listed above
b. Yes, I ate one of these foods once
c. Yes, I ate one of these foods twice
d. Yes, I ate one of these foods 3 times or more

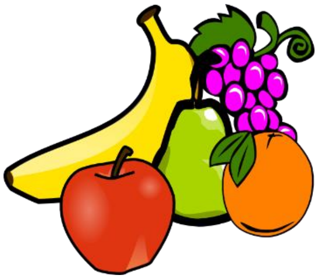

15. Yesterday, did you drink natural fruit juice?

a. No, I did not eat any of the foods listed above
b. Yes, I ate one of these foods once
c. Yes, I ate one of these foods twice
d. Yes, I ate one of these foods 3 times or more

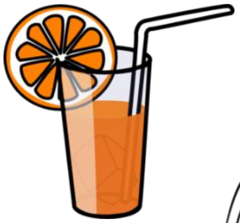

16. Yesterday, did you drink commercial fruit juice?

a. No, I did not eat any of the foods listed above
b. Yes, I ate one of these foods once
c. Yes, I ate one of these foods twice
d. Yes, I ate one of these foods 3 times or more

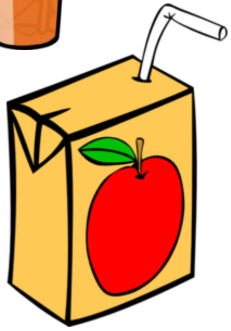

17. Yesterday, did you drink soft drinks (Pepsi, 7up, Miranda, Sprite, Coca Cola, Fanta…)?

a. No, I did not eat any of the foods listed above
b. Yes, I ate one of these foods once
c. Yes, I ate one of these foods twice
d. Yes, I ate one of these foods 3 times or more

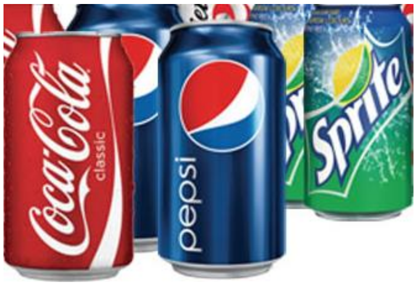

18. Yesterday, did you drink <u>diet</u> soft drinks (Pepsi diet, 7up diet, Coca Cola diet, …)?

a. No, I did not eat any of the foods listed above
b. Yes, I ate one of these foods once
c. Yes, I ate one of these foods twice
d. Yes, I ate one of these foods 3 times or more

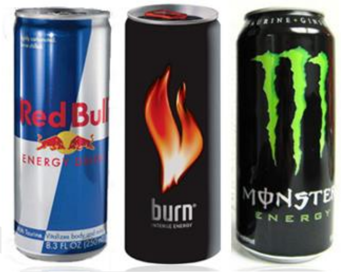

19. Yesterday, did you drink energy drinks (Red Bull, AMP, …)?

a. No, I did not eat any of the foods listed above
b. Yes, I ate one of these foods once
c. Yes, I ate one of these foods twice
d. Yes, I ate one of these foods 3 times or more

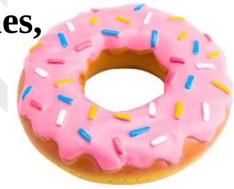

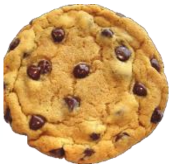

20. Yesterday, did you eat any sweets such as sweet rolls, cookies, cakes, pies, brownies, cheesecake, doughnuts?

a. No, I did not eat any of the foods listed above
b. Yes, I ate one of these foods once
c. Yes, I ate one of these foods twice
d. Yes, I ate one of these foods 3 times or more

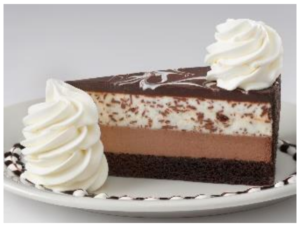

21. Yesterday, did you eat any Arabic sweets (namoura, knefeh, halewet el jibn, znood el sitt…)?

a. No, I did not eat any of the foods listed above
b. Yes, I ate one of these foods once
c. Yes, I ate one of these foods twice
d. Yes, I ate one of these foods 3 times or more

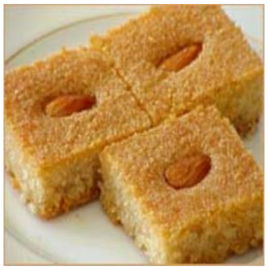

22. Yesterday, did you have breakfast?

a. Yes b. No

23. Yesterday, how many meals did you eat (meals include breakfast, lunch, and dinner)?

a. I did not have any meal
b. I had 1 meal
c. I had 2 meals
d. I had 3 or more meals

24. **Yesterday, did you have a snack? A snack is a food or drink (except for water) that you eat or drink between meals.**
   a. No, I did not eat any of the foods listed above
   b. Yes, I ate one of these foods once
   c. Yes, I ate one of these foods twice
   d. Yes, I ate one of these foods 3 times or more

25. **Yesterday, how many times did you eat food from outside of your house? (restaurants, fast food restaurants, pizza places and cafeterias)**
   a. No, I did not eat outside of the house
   b. Yes, I had 1 meal
   c. Yes, I had 2 meals
   d. Yes, I had 3 meals or more

26. **Do you practice physical activity regularly?**
   a. Yes   b. No

27. **How many hours per week do you exercise?**
   a. 0   b. 1-2   c. 3-4   d. >5

28. **How many hours per day do you usually spend playing video games, watching TV, using tablets and chatting on the phone?**
   a. 0hour b. 1hour c. 2hours d. 3hours e. 4hours f. 5hours g. 6hours h. >6hours

29. **Do you smoke? (cigarettes and narjileh are included)**
   a. Yes   b. No   c. Used to smoke

Adapted from SPANS 2010

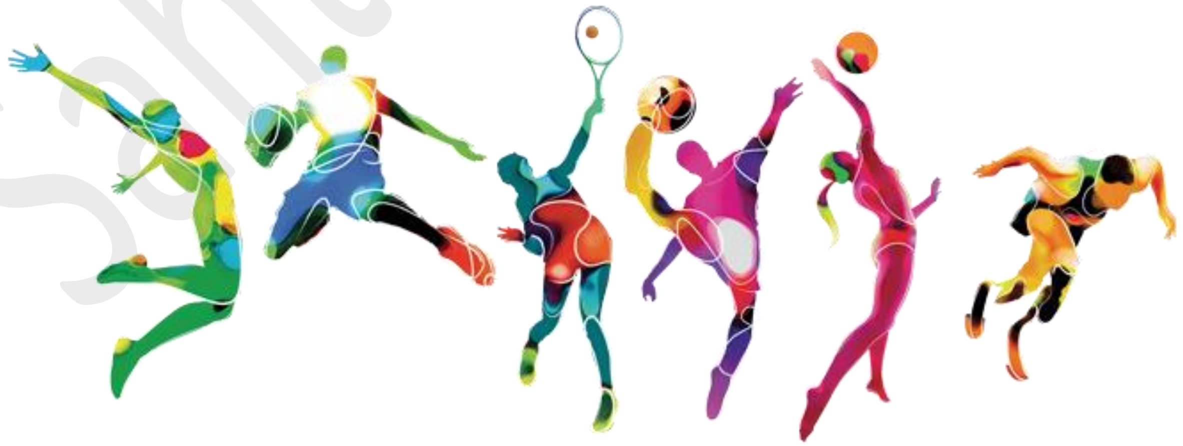

Sahtak bi Sahnak
