# Supplementary material for: Development of Dietary Knowledge and Adherence Questionnaires for Lebanese Adolescents and Their Parents
Source: Int J Environ Res Public Health. 2019 Dec 24;17(1):147. doi: 10.3390/ijerph17010147 (PMC6982025; doi:10.3390/ijerph17010147)

Dietary Adherence Questionnaire

حضرة الأهالي الأعزاء،

- ألرجاء تعبئة الإستبيان التالي بشأن العادات الغذائية والرياضة كجزء من بحث علمي تجريه جامعة ماسترشت في هولندا خلال العام الدراسي 2016-2017.
- تهدف هذه الدراسة إلى تقييم السلوك الغذائي لأهالي المراهقين اللبنانيين وأولادهم.
- سيطلب منكم الإجابة على 29 سؤال حول التقيد بالارشادات الغذائية، وذلك لمدة 20 دقيقة.
- يمكنك التوقف عن المشاركة في هذا البحث خلال اي وقت. إن المشاركة في هذا المشروع هي طوعية.
- ستكون جميع الإستبيانات مجهولة الهوية، وبالتالي لن يتم الأفصاح عن اية اجوبة.
- للمزيد من المعلومات، لا تتردد بطرح الأسئلة.
- من خلال تعبئة هذا الإستبيان، إنك توافق على المشاركة في هذا المشروع.

شكرً لدعمكم.

- **الرقم:** ______________________
- **العمر:** _______
- **شهر الولادة:**

○ كانون الثاني	○ شباط	○ اذار	○ نيسان
○ ايار	○ حزيران	○ تموز	○ اب
○ ايلول	○ تشرين الاول	○ تشرين الثاني	○ كانون الاول

- **سنة الولادة:**
- **رجل/امرأة:**

○ رجل ○ امرأة

العنوان (المدينة/القرية): ___________

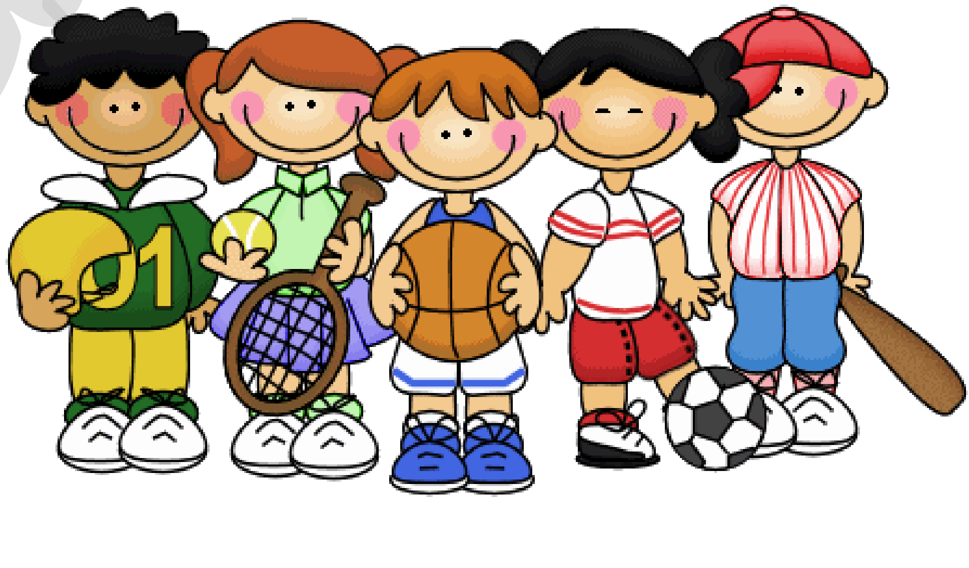

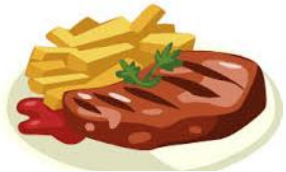

1. **ألبارحة، هل تناولت احدى الأطعمة التالية: لحم البقر(مثل اللحمة المفرومة في اليخاني)، سفينة الدجاج (غير مقلية/مشوية أو مسلوقة) أو السمك (الغير مقلي/ مشوية أو مسلوقة)؟**
   a. لا، لم أتناول أي من الأطعمة المذكورة
   b. نعم، تناولت احدى هذه الأطعمة مرة واحدة
   c. نعم، تناولت احدى هذه الأطعمة مرتان
   d. نعم، تناولت احدى هذه الأطعمة ثلاث مرات أو أكثر

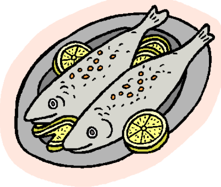

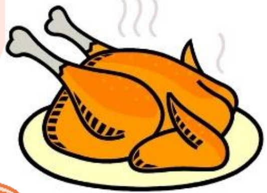

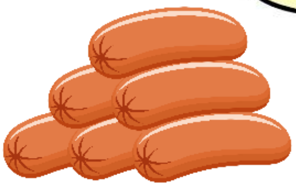

2. **ألبارحة، هل تناولت احدى الأطعمة التالية: دجاج مقلي أو دجاج ناغيتس أو سمك مقلي أو هوت دوغ أو سجق أو مرتديلا أو جانبون؟**
   a. لا، لم أتناول أي من الأطعمة المذكورة
   b. نعم، تناولت احدى هذه الأطعمة مرة واحدة
   c. نعم، تناولت احدى هذه الأطعمة مرتان
   d. نعم، تناولت احدى هذه الأطعمة ثلاث مرات أو أكثر

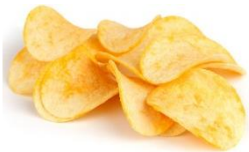

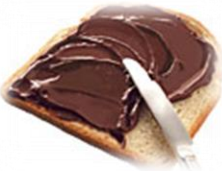

3. **ألبارحة، هل تناولت بطاطا تشيبس أو كريمة الشوكولا؟**
   a. لا، لم أتناول أي من الأطعمة المذكورة
   b. نعم، تناولت احدى هذه الأطعمة مرة واحدة
   c. نعم، تناولت احدى هذه الأطعمة مرتان
   d. نعم، تناولت احدى هذه الأطعمة ثلاث مرات

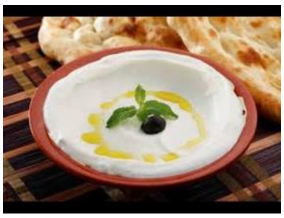

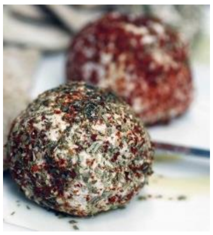

4. **ألبارحة، هل تناولت احدى الأطعمة التالية: لبنة، شنكليش، قريشة؟**
   a. لا، لم أتناول أي من الأطعمة المذكورة
   b. نعم، تناولت احدى هذه الأطعمة مرة واحدة
   c. نعم، تناولت احدى هذه الأطعمة مرتان
   d. نعم، تناولت احدى هذه الأطعمة ثلاث مرات أو أكثر

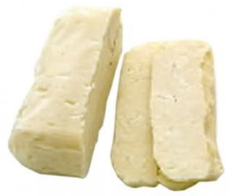

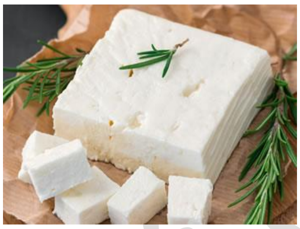

5. **ألبارحة، هل تناولت احدى الأجبان التالية: جبنة موزاريلا، فيتا، عكاوي، بلدية، جبنة خضرا؟**
   a. لا، لم أتناول أي من الأطعمة المذكورة
   b. نعم، تناولت احدى هذه الأطعمة مرة واحدة
   c. نعم، تناولت احدى هذه الأطعمة مرتان
   d. نعم، تناولت احدى هذه الأطعمة ثلاث مرات أو أكثر

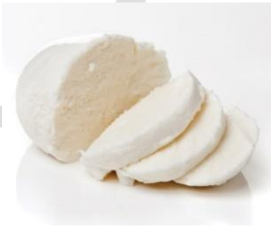

6. **ألبارحة، هل تناولت احدى الأطعمة التالية: جبنة شيدار، غروييرgruyere، جبنة ايدامedam، جبنة ماعز، جبنة غودا، جبنة بارميزانparmesan أو روكفورroquefort، قشقوان، كشك؟**
   a. لا، لم أتناول أي من الأطعمة المذكورة
   b. نعم، تناولت احدى هذه الأطعمة مرة واحدة
   c. نعم، تناولت احدى هذه الأطعمة مرتان
   d. نعم، تناولت احدى هذه الأطعمة ثلاث مرات أو أكثر

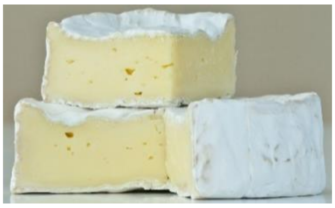

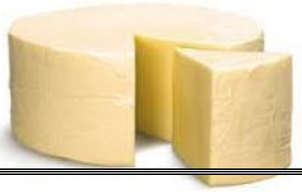

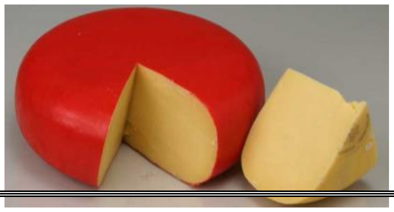

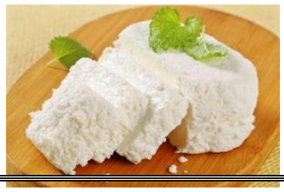

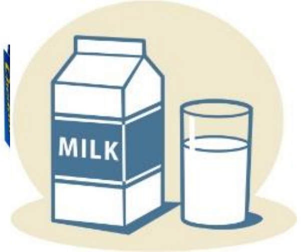

7. ألبارحة، هل تناولت الحليب أو اللبن؟

a. لا، لم أتناول أي من الأطعمة المذكورة
b. نعم، تناولت احدى هذه الأطعمة مرة واحدة
c. نعم، تناولت احدى هذه الأطعمة مرتان
d. نعم، تناولت احدى هذه الأطعمة ثلاث مرات أو أكثر

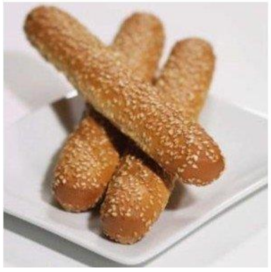

8. ألبارحة، هل تناولت الحليب القليل أو الخالي من الدسم أو اللبن الخالي من الدسم؟

a. لا، لم أتناول أي من الأطعمة المذكورة
b. نعم، تناولت احدى هذه الأطعمة مرة واحدة
c. نعم، تناولت احدى هذه الأطعمة مرتان
d. نعم، تناولت احدى هذه الأطعمة ثلاث مرات أو أكثر

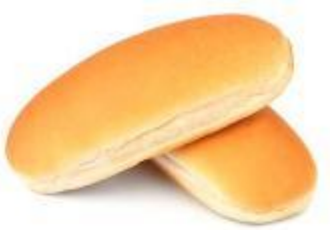

9. ألبارحة، هل تناولت الخبز العربي الابيض أو الكعك أو الخبز الفرنجي الابيض؟

a. لا، لم أتناول أي من الأطعمة المذكورة
b. نعم، تناولت احدى هذه الأطعمة مرة واحدة
c. نعم، تناولت احدى هذه الأطعمة مرتان
d. نعم، تناولت احدى هذه الأطعمة ثلاث مرات

10. ألبارحة، هل تناولت الخبز الاسمر أو خبز القمح الكامل أو خبز الشوفان أو التورتيلاtortilla؟

a. لا، لم أتناول أي من الأطعمة المذكورة
b. نعم، تناولت احدى هذه الأطعمة مرة واحدة
c. نعم، تناولت احدى هذه الأطعمة مرتان
d. نعم، تناولت احدى هذه الأطعمة ثلاث مرات أو أكثر

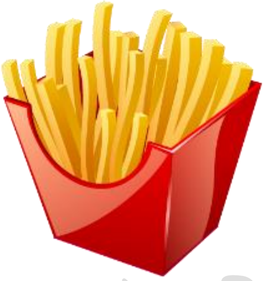

11. ألبارحة، هل تناولت البطاطا المقلية؟

a. لا، لم أتناول أي من الأطعمة المذكورة
b. نعم، تناولت احدى هذه الأطعمة مرة واحدة
c. نعم، تناولت احدى هذه الأطعمة مرتان
d. نعم، تناولت احدى هذه الأطعمة ثلاث مرات أو أكثر

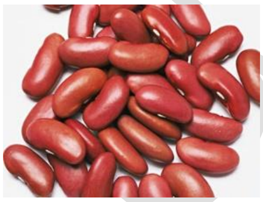

12. ألبارحة، هل تناولت البقوليات كالفاصوليا أو العدس أو الفول؟

a. لا، لم أتناول أي من الأطعمة المذكورة
b. نعم، تناولت احدى هذه الأطعمة مرة واحدة
c. نعم، تناولت احدى هذه الأطعمة مرتان
d. نعم، تناولت احدى هذه الأطعمة ثلاث مرات أو أكثر

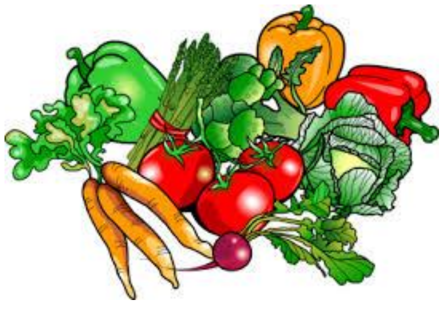

13. ألبارحة، هل تناولت الخضار؟ (البطاطا لا تحتسب)

a. لا، لم أتناول أي من الأطعمة المذكورة
b. نعم، تناولت احدى هذه الأطعمة مرة واحدة
c. نعم، تناولت احدى هذه الأطعمة مرتان
d. نعم، تناولت احدى هذه الأطعمة ثلاث مرات أو أكثر

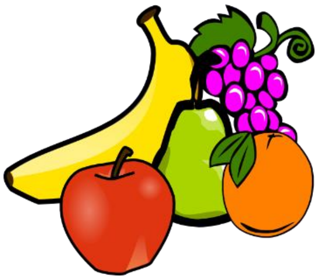

14.ألبارحة، هل تناولت الفاكهة الطازجة؟ (عصير الفاكهة والفاكهة المجففة لا تحتسب)

a. لا، لم أتناول أي من الأطعمة المذكورة
b. نعم، تناولت احدى هذه الأطعمة مرة واحدة
c. نعم، تناولت احدى هذه الأطعمة مرتان
d. نعم، تناولت احدى هذه الأطعمة ثلاث مرات أو أكثر

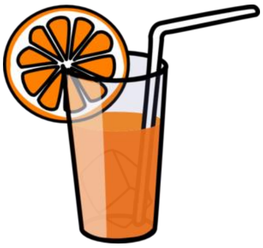

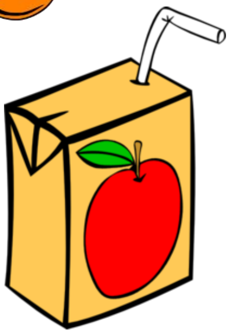

15.ألبارحة، هل شربت عصير الفاكهة الطبيعي؟

a. لا، لم أتناول أي من الأطعمة المذكورة
b. نعم، تناولت احدى هذه الأطعمة مرة واحدة
c. نعم، تناولت احدى هذه الأطعمة مرتان
d. نعم، تناولت احدى هذه الأطعمة ثلاث مرات أو أكثر

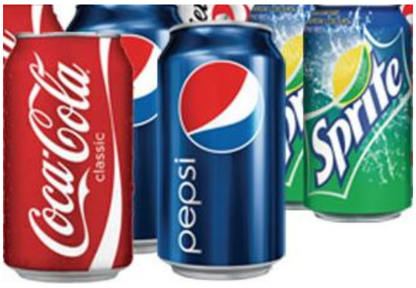

16.ألبارحة، هل شربت عصير الفاكهة الإصطناعي؟

a. لا، لم أتناول أي من الأطعمة المذكورة
b. نعم، تناولت احدى هذه الأطعمة مرة واحدة
c. نعم، تناولت احدى هذه الأطعمة مرتان
d. نعم، تناولت احدى هذه الأطعمة ثلاث مرات أو أكثر

17.ألبارحة، هل شربت المشروبات الغازية (كالبيبسي والسفن اب والميرندا والكوكا كولا وغيرها)؟

a. لا، لم أتناول أي من الأطعمة المذكورة
b. نعم، تناولت احدى هذه الأطعمة مرة واحدة
c. نعم، تناولت احدى هذه الأطعمة مرتان
d. نعم، تناولت احدى هذه الأطعمة ثلاث مرات أو أكثر

18.ألبارحة، هل شربت المشروبات الغازية الدايت (كالبيبسي دايت والسفن اب دايت وغيرها)؟

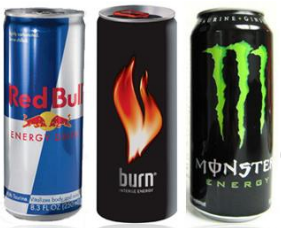

a. لا، لم أتناول أي من الأطعمة المذكورة
b. نعم، تناولت احدى هذه الأطعمة مرة واحدة
c. نعم، تناولت احدى هذه الأطعمة مرتان
d. نعم، تناولت احدى هذه الأطعمة ثلاث مرات أو أكثر

19.ألبارحة، هل شربت مشروب الطاقة (Red Bull, AMP...) ؟

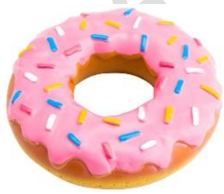

a. لا، لم أتناول أي من الأطعمة المذكورة
b. نعم، تناولت احدى هذه الأطعمة مرة واحدة
c. نعم، تناولت احدى هذه الأطعمة مرتان
d. نعم، تناولت احدى هذه الأطعمة ثلاث مرات أو أكثر

20.ألبارحة، هل تناولت الحلويات كالكوكيز cookies والبراونيز brownies والتشيزكيك cheesecake والدونت donut؟

a. لا، لم أتناول أي من الأطعمة المذكورة
b. نعم، تناولت احدى هذه الأطعمة مرة واحدة
c. نعم، تناولت احدى هذه الأطعمة مرتان
d. نعم، تناولت احدى هذه الأطعمة ثلاث مرات أو أكثر

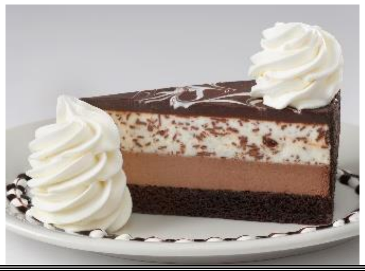

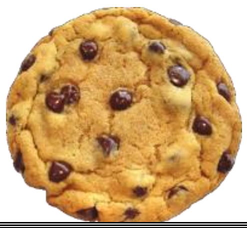

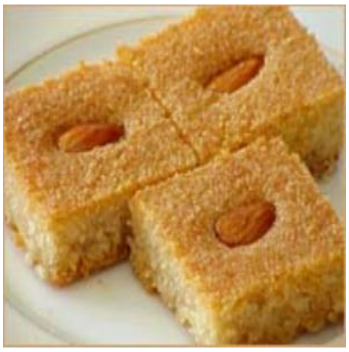

21.ألبارحة، هل تناولت الحلويات العربية (كالنمورة والكنافة وحلاوة الجبن ,...)؟

a. لا، لم أتناول أي من الأطعمة المذكورة
b. نعم، تناولت احدى هذه الأطعمة مرة واحدة
c. نعم، تناولت احدى هذه الأطعمة مرتان
d. نعم، تناولت احدى هذه الأطعمة ثلاث مرات أو أكثر

22.ألبارحة، هل تناولت الفطور؟

a. نعم b. لا

23.ألبارحة، كم وجبة تناولت (فطور/ غداء/ عشاء)؟

a. لم أتناول اية وجبة b. تناولت وجبة واحدة c. تناولت وجبتين d. تناولت ثلاث وجبات

24.ألبارحة، كم وجبة خفيفة أو سناك تناولت؟ (السناك هو طعام أو شراب تتناوله بين الوجبات الرئيسية/ الماء لا يعد سناك)

a. لم أتناول اية وجبة b. تناولت وجبة واحدة c. تناولت وجبتين d. تناولت ثلاث وجبات خفيفة أو أكثر

25.ألبارحة، كم مرة تناولت طعاماً خارج المنزل (في احدى المطاعم، مطعم fast food، مطعم بيتزا، كافيتيريا)؟

a. لم أتناول اية وجبة خارج البيت b. تناولت وجبة واحدة
c. تناولت وجبتين d. تناولت ثلاثة وجبات أو أكثر

26.هل تمارس الرياضة بانتظام؟

a. نعم b. لا

27.كم ساعة تمارس الرياضة أسبوعياً؟

a. 0 b. 1 c. 2-4 d. 5 أو أكثر

28.كم ساعة تمضي يومياً أمام شاشة التلفاز أو التلفون أو التابليت؟

a. 0 b. 1 c. 2 d. 3 e. 4 f. 5 g. 6 ساعات أو أكثر

29.هل تدخن (سجائر/ نرجيلة)؟

a. نعم b. لا c. توقفت عن التدخين

Adapted from SPANS 2010

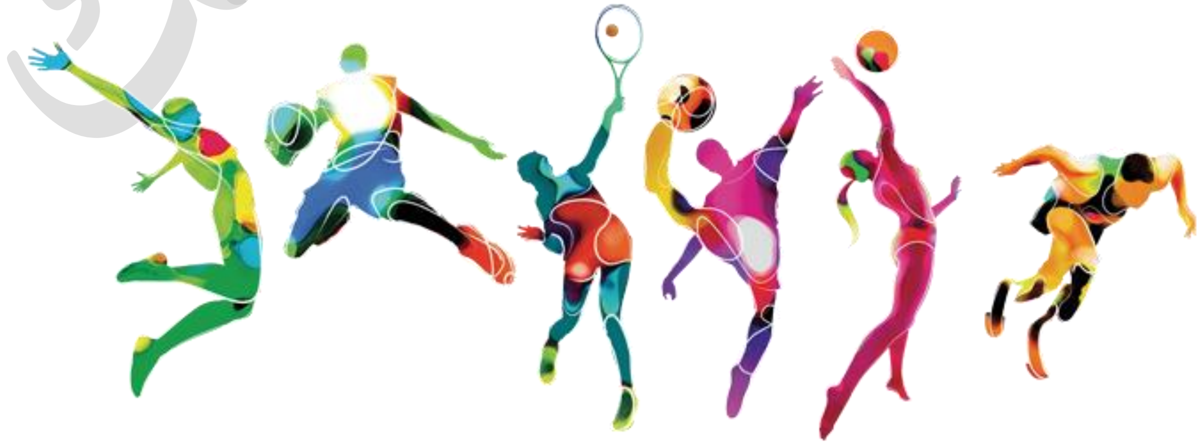

Supplement: Supplementary file 1 [file ijerph-17-00147-s001.zip › Supplementary material - S8 - DAQ parents Ar.pdf]
